# Comparison of Salt Tolerance in *Soja* Based on Metabolomics of Seedling Roots

**DOI:** 10.3389/fpls.2017.01101

**Published:** 2017-06-23

**Authors:** Mingxia Li, Rui Guo, Yang Jiao, Xiaofei Jin, Haiyan Zhang, Lianxuan Shi

**Affiliations:** ^1^School of Life Sciences, Northeast Normal UniversityChangchun, China; ^2^Key Laboratory of Dryland Agriculture, Institute of Environment and Sustainable Development in Agriculture, Chinese Academy of Agricultural SciencesBeijing, China; ^3^Jilin Province Crop Breeding Center of New VarietiesChangchun, China

**Keywords:** *soja*, roots, metabolomics, salt stress, salt tolerance

## Abstract

Soybean is an important economic crop that is continually threatened by abiotic stresses, especially salt stress. Wild soybean is an important germplasm resource for the breeding of cultivated soybean. The root system plays a very important role in plant salt tolerance. To explore the salt tolerance-related mechanisms among *Soja*, we have demonstrated the seedling roots' growth and metabolomics in wild soybean, semi-wild soybean, and cultivated soybean under two types of salt stress by using gas chromatography-mass spectrometry. We characterized 47 kinds of differential metabolites under neutral salt stress, and isoleucine, serine, l-allothreonine, glutamic acid, phenylalanine, asparagines, aspartic acid, pentadecanoic acid, lignoceric acid, oleic acid, galactose, tagatose, d-arabitol, dihydroxyacetone, 3-hydroxybutyric acid, and glucuronic acid increased significantly in the roots of wild soybean seedlings. However, these metabolites were suppressed in semi-wild and cultivated soybeans. Amino acid, fatty acid, sugars, and organic acid synthesis and the secondary metabolism of antioxidants increased significantly in the roots of wild soybean seedling. Under alkaline salt stress, wild soybean contained significantly higher amounts of proline, glutamic acid, aspartic acid, l-allothreonine, isoleucine, serine, alanine, arachidic acid, oleic acid, cis-gondoic acid, fumaric acid, l-malic acid, citric acid, malonic acid, gluconic acid, 5-methoxytryptamine, salicylic acid, and fluorene than semi-wild and cultivated soybeans. Our study demonstrated that carbon and nitrogen metabolism, and the tricarboxylic acid (TCA) cycle and receiver operating characteristics (especially the metabolism of phenolic substances) of the seedling roots were important to resisting salt stress and showed a regular decreasing trend from wild soybean to cultivated soybean. The metabolomics's changes were critical factors in the evolution of salt tolerance among *Soja*. This study provides new insights into salt tolerance in soybean, and presents quantitative parameters for a salt tolerant soybean breeding system, which is conducive to the rational use and protection of wild soybean resources.

## Introduction

Soybean is an important source of protein and fat in the human diet, as well as being an important livestock feed and industrial raw material (He and Chen, 2013). *Soja* includes wild soybean (*Glycine soja*), semi-wild soybean (*Glycine gracilis*), and cultivated soybean (*Glycine max*), which are phylogenetically related. Wild soybean can better adapt to a variety of adverse environments (Tuyen et al., 2010). Semi-wild soybean is a transition type in the evolution of *Soja*, having a physiological metabolism close to that of wild soybean and a phenotype closer to that of cultivated soybean (Shi et al., 2016). The cultivated soybean has been bred by artificial selection and domestication from wild soybean. However, cultivated soybean is a typical glycophyte in which growth, quality, and yield are significantly reduced under salt stress (Wang and Li, 2011).

Soil salinity and alkalinity seriously affect plant growth and development, which negatively influences the development of agriculture and animal husbandry (Guo et al., 2009). Alkaline salts (NaHCO_3_ and Na_2_CO_3_) are more destructive to plants than neutral salts (NaCl and Na_2_SO_4_). While neutral salts stress in a soil generally involves osmotic stress and ion-induced injury, there is an added high pH effect with alkaline salts stress (Munns, 2002; Guo et al., 2009; Yang et al., 2012; Rahman et al., 2016). To date, the research of resistance to salt stress of plants has focused on genomics, transcriptomics, and proteomics (Zhang et al., 2012; Qi et al., 2014); however, these techniques cannot fully determine the changes in metabolites and their metabolic pathways comprehensively in plants under salt stress (Sumner et al., 2003). Therefore, metabolomics has rapidly developed to provide information downstream of genetic responses to biotic and abiotic stresses, especially in plant salt-tolerance-related research (Dona et al., 2016). For example, Wu et al. analyzed the metabolic differences between wild and cultivated barley under salt stress, osmotic regulation is the most basic mechanism for improving barley salt tolerance, and the accumulation of sodium in the tissues and the rearrangement of nutrients and metabolites improved the salt tolerance of barley (Wu et al., 2013a,b). Hu et al. (2014) studied *Poa pratensis*, in which salt stress is mainly related to amino acids and sugars, while the alkaline stress is mainly related to the accumulation of sugar acid, organic acid, and the tricarboxylic acid (TCA) cycle. Kim et al.'s (2007) studies on *Arabidopsis* showed that under salt stress the secondary metabolic pathways in lignin production and glycinebetaine biosynthesis were induced. Lu et al. (2013) studied the mechanism of salt tolerance in soybean based on metabolomics and concluded that the salt tolerance of soybean is mainly based on the synthesis of compatible solutes, the induction of reactive oxygen species (ROS) scavengers, the modification of cell membranes, and the induction of plant hormones. As far as we know, there is limited research based on metabolomics that reveals the different metabolic reactions of wild soybean, semi-wild soybean, and cultivated soybean in the same area under neutral and alkaline salt stresses. Notably there were statistically significant differences in the metabolism of different organs in plants, and the small molecular metabolites were different between roots and leaves under Salt Stress. It is of great importance to understand and improve the salt tolerance mechanism in soybean.

Here, we described the metabolomics of wild, semi-wild, and cultivated soybeans seedling roots under salt stress using gas chromatography-mass spectrometry (GC-MS) analysis technology. The aims of this study were as follows: (1) To investigate the differences in growth and root metabolomics of *Soja* under neutral salt and alkaline salt stresses; and (2) To discuss the differences in the salt-tolerance mechanisms among wild, semi-wild, and cultivated soybeans under the two types of salt stress. The physiological mechanisms related to the change in root' salt tolerance during the domestication of soybean is explained, which provides a quantitative parameter system for the cultivation of salt-tolerant soybean. This study provided a theoretical basis for the mining of the salt-tolerant genes of soybean and the sustainable production of soybean. It will also improve the utilization of saline land, improve China's ecological environment and promote the sustainable development of China's agriculture.

## Materials and methods

### Experimental materials and growing conditions

The experimental materials are wild soybean (W; Huinan06116), semi-wild soybean (S), and cultivated soybean (M; Jinong24). They were provided by Jilin Province Crop Breeding Center of New Varieties. Three seeds were sown in 14-cm diameter plastic pots, each containing 2.5 kg of washed sand. Seedlings were watered daily with 1X strength Hoagland's nutrient solution. All of the pots were placed outdoors but were sheltered from the rain. The night time temperature was 18.5 ± 1.5°C, the daytime temperature was 26 ± 2°C, and the humidity was 60 ± 5%. After emergence, one seedling per pot was selected based on its uniform growth. In this study, there were two types of salt stress neutral and alkaline. Each stress concentration was used in 8 pots parallel treatments, totaling 84 pots, and 48 pots were used to determine the basal biomass, while 36 pots were used for the metabolomics analysis.

### Stress treatment

The stress treatments used were divided into neutral salt stress (NaCl and Na_2_SO_4_, at a 1:1 molar ratio, 45 mmol·L^−1^ Na^+^, pH 6.71) and alkaline salt stress (Na_2_CO_3_ and NaHCO_3_, at a 1:1 molar ratio, 45 mmol·L^−1^ Na^+^, pH 9.77). In the control group, soybeans were cultivated with 1X Hoagland's nutrient solution, and the salt-treated groups were exposed to neutral-salt stress and to alkaline salt stress. First, these seedlings were treated with two types of stress solutions containing 15 mmol·L^−1^ Na^+^ for the first 2 days, and then 30 mmol·L^−1^ Na^+^ for the next 2 days, to allow seedlings to gradually adapt to salt stress. Finally, the three soybean cultivars were each treated separately with the two treatments containing 45 mmol·L^−1^ Na^+^ for 14 days. In addition, the growth status of the experimental materials was recorded every day. At the end of the treatment, roots without root nodules were harvested from four clones (biological replicates) from each treatment of each soybean genotype and used as test materials. The rest of the clones were used to measure growth indices under each treatment. The collected root samples were immediately ground and frozen in liquid nitrogen, and then stored at −80°C until further use.

### Measurement of growth parameters, and the extraction and analysis of metabolites

After the soybean plants were harvested, shoot heights, root lengths, and the dry weight (DWs) of the shoots and roots were measured (Shi et al., 2015), and the relative growth rates (RGRs) of seedlings were determined according to Kingsbury et al. (1984) as follows: RGR = (In DW1 − In DW0)/(*t*_2_ − *t*_1_), where W0 represents the first DW, W1 represents the last DW, and *t*_2_ − *t*_1_ represents the total treatment's duration).

The root samples were pooled, homogenized and 100 ± 5 mg of the plant materials were transferred to an Eppendorf tube (2 mL). Plant materials were extracted with 0.5 mL of extraction liquid (methanol: chloroform, 3:1, v: v) and 60 μL of ribitol (0.2 mg·mL^−1^ stock in H_2_O) as an internal standard were added. Using a Thermomixer (Eppendorf AG, Hamburg, Germany) at 70°C and 950 rpm for 10 min, the samples were extracted. Subsequently, the tubes were centrifuged at 12,000 rpm at 4°C for 10 min. Then, 0.4 mL of the supernatant was transferred into a 2 mL-GC-MS glass vial, and samples were dried in a vacuum concentrator at 30°C for 2 h. Then, each sample was dissolved in 80 μL of methoxamine hydrochloride (20 mg·mL^−1^ in pyridine). The 80 μL aliquot of the supernatant was further derivatized by methoxyamination with a 20 mg·mL^−1^ solution of methoxyamine hydrochloride in pyridine. They were dried in a vacuum concentrator, and placed in an oven (MKX-J1-10, Qingdao Makewave Microwave Technology Co. Ltd., Qingdao, China) adjusted to 37°C for 2 h. Subsequently, samples were derivatized with trimethylsilylation with *N*-methyl-*N*-(tri methylsilyl) trifluoroacetamide at 70°C for 1 h. When the temperature fell to room temperature, all of the samples were used for GC-MS analysis using an Agilent 7890 GC system coupled to a Pegasus HT time-of-flight MS. A 1-μL aliquot of the analyte was injected in splitless mode. Helium was used as the carrier gas, with a flow rate of 1 mL·min^−1^ after the front inlet purge flow was 3 mL·min^−1^. The column temperature was maintained at 70°C during the first 2 min and then increased to 300°C at a rate of 10°C min^−1^ and maintained for 10 min. The total analysis time was 35 min. The injector temperature was 280°C and transfer line 280 was used. Ionization in the ion source at a temperature of 220°C was coupled with the electron energy of 70 eV. Mass spectra were recorded in the range 50–650 m·z^−1^.

### Data processing and multivariate data analysis

The data were acquired and pre-processed using the manufacturer's ChromaTOF software (versions 2.12, 2.22, and 3.34; LECO, St. Joseph, MI, USA). The metabolites were identified by searching the commercial EI-MS and the FiehnLib libraries. Then, at least 80% of missing values were removed and replaced with a small value, which was half of the minimum positive value in the original data. The data were filtered using the interquartile range, and the total mass of the signal integration area was normalized for each sample. The normalized data were fed into the SIMCA-P 13.0 software package (Umetrics, Umea, Sweden) for variable importance in the projection values (VIP) obtained through partial least squares discriminant and orthogonal partial least squares discriminant analyses. The principal component, partial least squares discriminant and orthogonal partial least squares discriminant analyses were performed using the SIMCA-P 13.0 software package (Umetrics). Additionally, differential metabolites were found using Student's *t*-test (*p* < 0.05) and VIP (VIP > 1), combined with similarity values >700. Subsequently, the metabolic pathway was constructed according to KEGG (http://www.genome.jp/kegg/), and the pathway was analyzed using MetaboAnalyst which is based on the change in metabolite concentration compared with the corresponding controls (Xia et al., 2012).

## Results

### Changes in growth performance among *Soja* under salt stress

**Figure 1:**
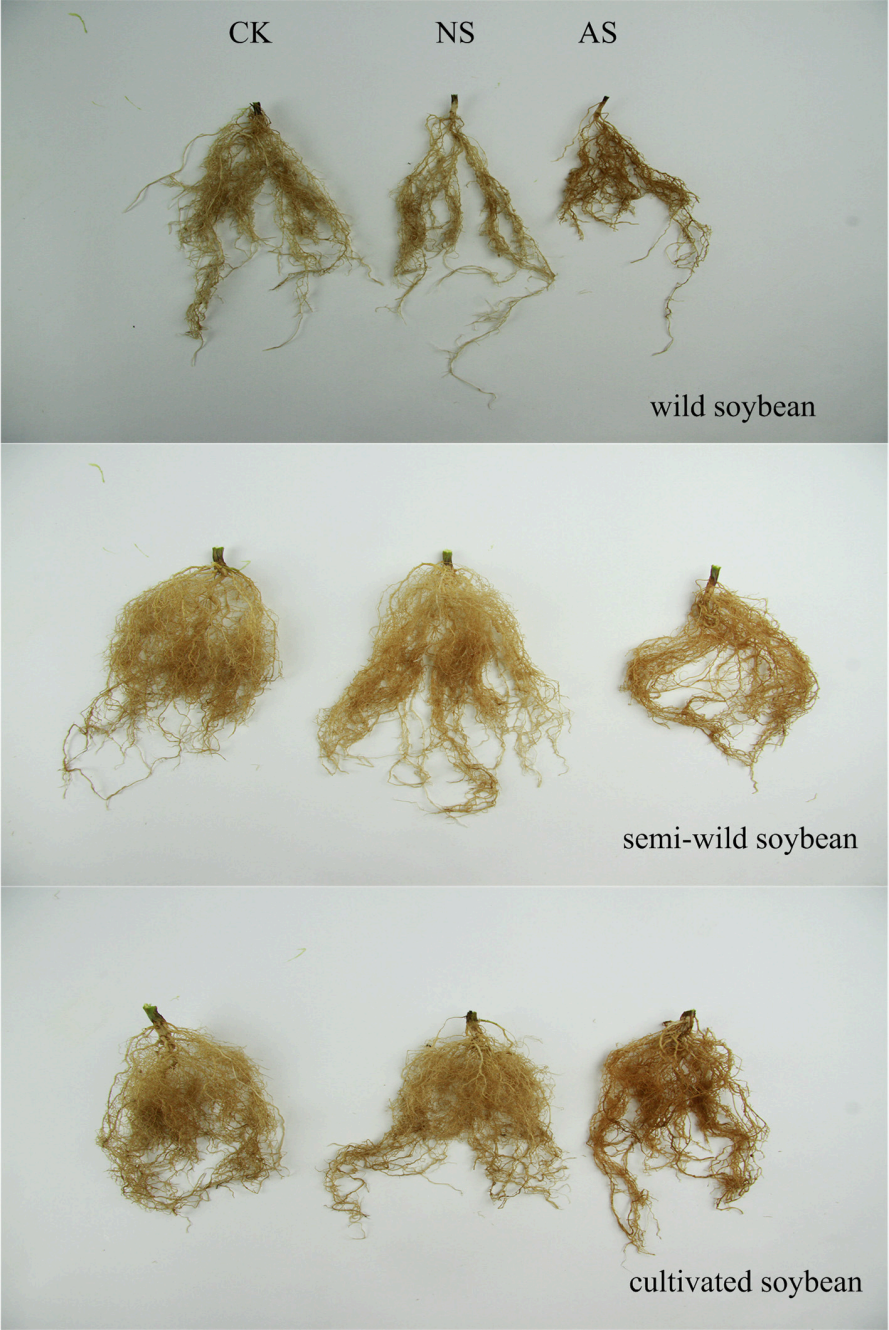
The growth performances of the three soybean genotypes under normal and two salt conditions. CK, control treatment; NS, neutral salt stress; AS, alkaline salt stress.

Seedling root growth and biomass were significantly inhibited in the three kinds of soybean when exposed to both kinds of salt stress (Table 1). The shoot heights and root lengths of the three soybean cultivars decreased significantly under both types of salt stress (*p* < 0.05), and the amplitude of the inhibition in cultivated soybean was higher than that in the wild soybean. The decrease in the DW of shoots and roots had the same trend as the shoot heights and root lengths, and reached significant levels (*p* < 0.05). In addition, the changes in the RGRs of the shoots and roots were similar. However, the root system suffered greater damages than the aboveground parts among *soja*. Furthermore, the damage to plants from alkaline salt stress was greater than that from the neutral salt stress. Compared with the control, the number of leaves and roots per plant decreased, the leaf area became smaller, the leaves turned yellow under both types of salt stress. All of these changes were severer in cultivated soybean than in semi-wild and wild soybean (Figure 1, Supplementary Figure 1).

**Table 1:** The growth performances among *soja* seedlings under normal and two types of salt stress.

**Experimental materials**	**Growth parameters**	**Treatments**			**Fold changes**${\text{log}}_{2}^{\left(\text{salt}/\text{control}\right)}$	
		**CK**	**NS**	**AS**	**NS**	**AS**
W	Shoot height (cm)	115.50	95.33	76.00	−0.28^*^	−0.60^*^
	Root length (cm)	19.50	17.25	16.00	−0.12^*^	−0.18^*^
	Dry weight of shoots (g)	2.21	2.16	1.88	−0.03	−0.23
	Dry weight of roots (g)	0.54	0.41	0.36	−0.40^*^	−0.58^*^
	RGR of shoots	0.06	0.05	0.04	−0.23	−0.47
	RGR of roots	0.05	0.03	0.02	−0.25^*^	−0.34^*^
S	Shoot height (cm)	89.00	74.21	58.67	−0.26^*^	−0.60^*^
	Root length (cm)	20.03	18.13	17.50	−0.14^*^	−0.19^*^
	Dry weight of shoots (g)	3.08	2.83	2.47	−0.12	−0.32
	Dry weight of roots (g)	0.78	0.56	0.48	−0.48^*^	−0.70^*^
	RGR of shoots	0.04	0.03	0.03	−0.24^*^	−0.49^*^
	RGR of roots	0.04	0.04	0.02	−0.25^*^	−0.82^*^
M	Shoot height (cm)	90.33	70.54	56.23	−0.36^*^	−0.68^*^
	Root length (cm)	17.50	15.14	14.23	−0.21^*^	−0.30^*^
	Dry weight of shoots (g)	3.37	3.31	2.93	−0.03	−0.20
	Dry weight of roots (g)	0.81	0.66	0.52	−0.30^*^	−0.64^*^
	RGR of shoots	0.04	0.03	0.03	−0.35^*^	−0.62^*^
	RGR of roots	0.02	0.01	0.01	−0.50^*^	−0.90^*^

*The data are the means from four biological replicates. The fold changes was calculated using the formula ${\text{log}}_{\text{2}}^{\left(salt/control\right)}$. Values were presented as the mean ± standard deviation of four biological replicates. CK, control treatment; NS, neutral-salt stress; AS, alkaline salt stress. W, wild soybean; S, semi-wild soybean; M, cultivated soybean*.

* *Significant difference at p < 0.05*.

### Metabolic profiles among *Soja* seedling' roots

**Figure 2:**
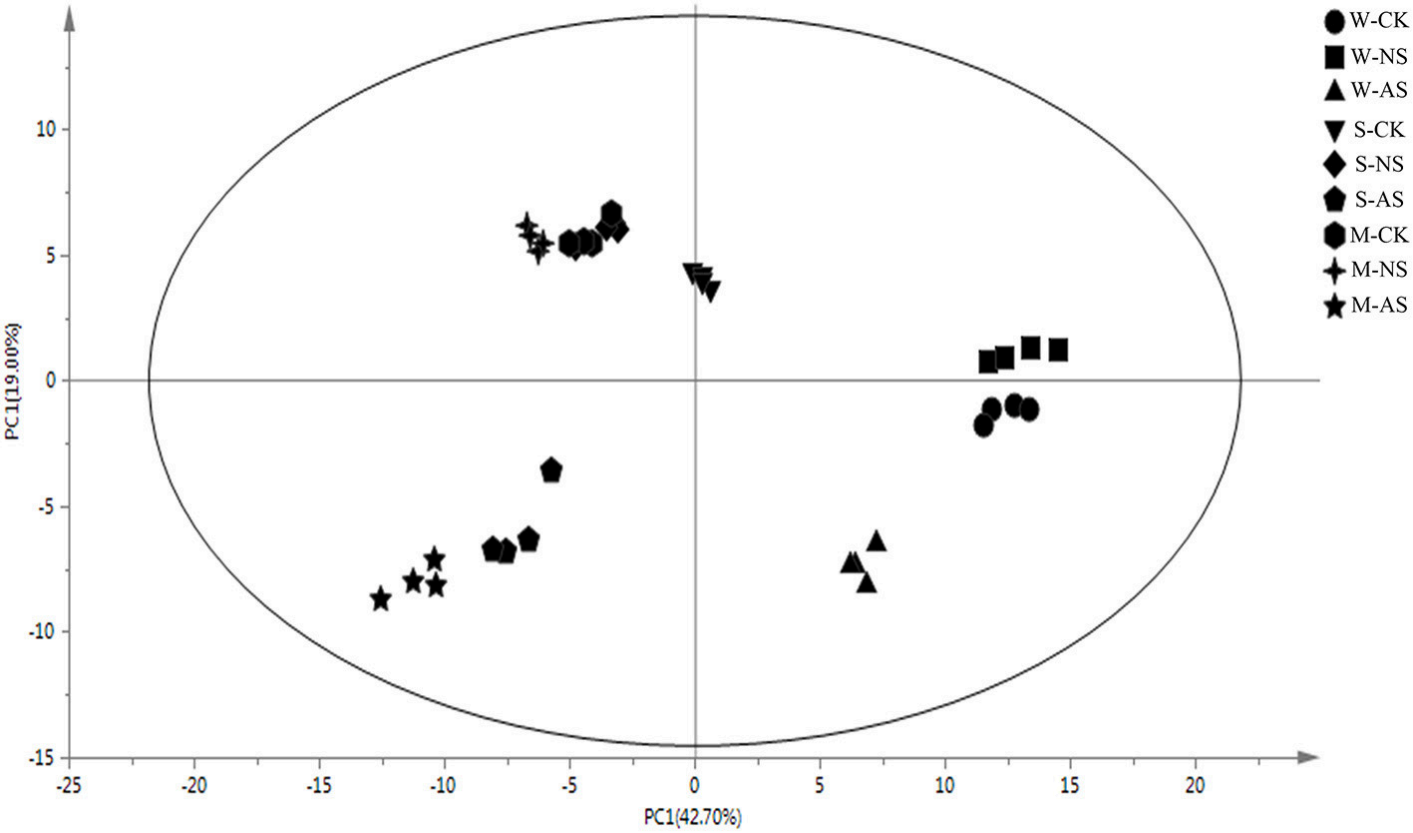
Principal component analysis (PCA) of metabolic profiles in the roots of W, S, and M under control and 45 mmol.L^−^1 neutral salt and alkaline salt stress (four biological replicates). W, wild soybean; S, semi-wild soybean; M, cultivated soybean; CK, control treatment; NS, neutral salt stress; AS, alkaline salt stress; PC1, the first principal component; PC2, the second principal component.

**Figure 3:**
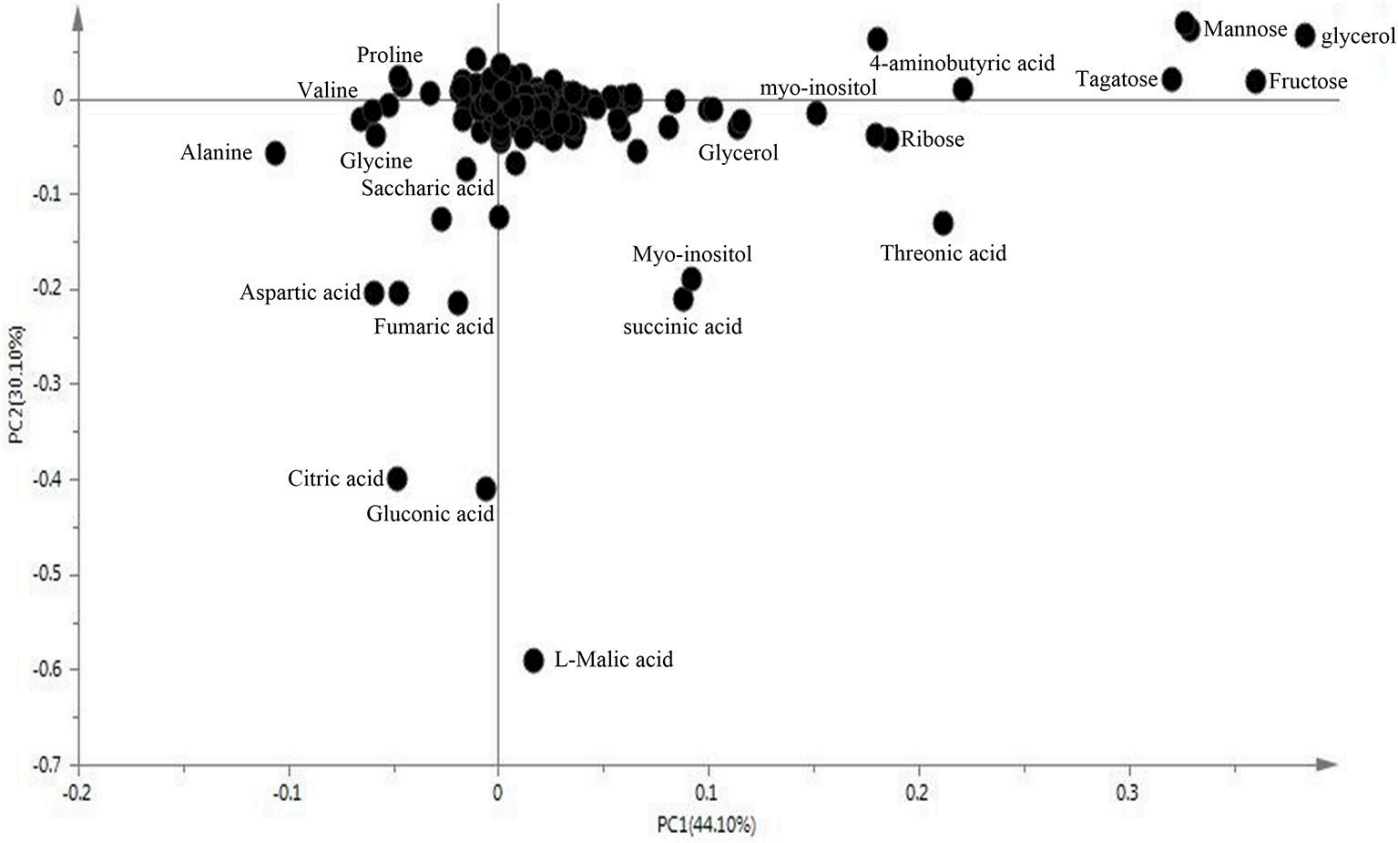
The loading plot of metabolites to the PC1 and PC2.

We examined metabolomics and metabolites profiles of *Soja* under normal and two types of salt stress conditions. By comparing the profile differences of soybean, we could distinguish differences in the salt tolerances among wild, semi-wild, and cultivated soybeans under two types of salt stress. To ensure the reliability of the experimental data and results, we established four parallel groups. The chromatograms of *Soja* seedling roots were obtained using the GC-MS analysis technique (Supplementary Table 1). There were significant differences between the peaks, which indicated that the experimental metabolomics analysis technology is suitable. Finally, the metabolites of soybean were analyzed under the two kinds of salt stress using the criteria *p* ≤ 0.05, similarity >700 and VIP >1. We focused on 69 metabolites, which were divided into the seven major categories of amino acids, fatty acids, sugar alcohols, carboxylic acids, TCA cycle intermediates, antioxidant substances, and nucleic acids. The principal component analysis analyzed by SIMCA-P 13.0 software revealed differences in the metabolites of different genotypes of *Soja* under both types of salt stress (Figures 2, 3; Supplementary Table 2). PC1 was 42.7%, with the main contribution coming from asparagine, eicosenoic acid, d-glyceric acid, glycerol, maltotriose, phytosphingosine, talose, glucose, fucose, ribose, mannose, fructose, xylitol, salicylic acid, putrescine, and hydroxylamine; PC2 was 19%, with the main contribution coming from aspartic acid, asparagine, pentadecanoic acid, sucrose, phytosphingosine, sitosterol, citric acid, fumaric acid, l-malic acid, galacturonic acid, and fluorene.

### Metabolic profiles among *Soja* seedling roots under normal conditions

Through metabolic profiling analysis, based on the PCs, we focused on the changes of 69 major metabolites in *Soja* seedling roots. These substances were divided into seven categories among them were 11 amino acids, 11 fatty acids 15 sugar and alcohols, 8 the TCA cycle related metabolites, 12 carboxylic acids, 3 nucleic acids, and 9 receiver operating characteristics (ROC; Supplementary Table 3). The metabolites of amino acid, fatty acid, sugar, and alcohols, ROC and TCA intermediates in *Soja* seedling roots showed significant differences. The contents of most amino acids in S and M were higher than those in W. The levels of proline, phenylalanine, glutamic acid, l-allothreonine, isoleucine, and serine, increased (*p* < 0.05) by 1.31-, 2.12-, 3.63-, 1.19-, 1.36-, and 2.30-fold, respectively, in S compared with W; and were increased (*p* < 0.05) to 2.11-, 2.50-, 3.63-, 1.51-, 1.95-, and 2.5-fold, respectively, in M compared with W. The contents of arachidonic acid, d-glyceric acid, and glycerol were significantly lower in S and M than in W. The three metabolites decreased (*p* < 0.05) by −1.58-, −0.85-, and −0.74-fold, respectively, in S compared with W, and −0.85-, −0.85-, and −0.92-fold, respectively, in M compared with W. The sugar alcohol contents in S and M seedling roots were mostly lower than in W, and, of these, significant reductions were seen in tagatose, fucose, ribose, mannitol, fructose, and xylitol. The contents of these metabolites decreased by −1.04-, −1.26-, −1.40-, −0.65-, −1.03-, and −1.27-fold those in S compared with W; and −0.84-, −0.88-, −0.74-, −1.87-, −0.73-, and −1.11-fold, respectively, in M compared with W (*p* < 0.05). The TCA cycle-related metabolites in S and M, including citramalic acid, succinic acid, glucose-1-phosphate, fumaric acid, l-malic acid, and citric acid, were lower than W. Succinic acid, fumaric acid, and l-malic acid decreased by −1.08-, −1.56-, and −1.26-fold, respectively, significantly lower in S compared with W; and −0.93-, −1.14-, and −0.99-fold, respectively, lower in M than W (*p* < 0.05). The contents of carboxylic acid in S and M seedling roots were lower than W, with significant reductions in 4-aminobutyric acid, threonic acid, and myristic acid. Their contents decreased by −0.80-, −1.34-, and −0.32-fold in S compared with W, and −1.38-, −1.05-, and −0.74-fold in M compared with W (*p* < 0.05). In addition, the materials related to ROC in S and M were lower than W, in which the flavonoid metabolites primuletin and the plant hormone metabolites salicylic acid, as well as polyamine hydroxylamine, were significantly decreased by −1.00-, −1.00-, and −2.00-fold in S, respectively; and −1.00-, −2.80-, and −2.00-fold in M, respectively, compared with W (*p* < 0.05). In addition to these metabolites, the contents of three nucleic acid metabolites, thymidine, thymine, and uracil, were lower in S and M than in W.

### Metabolic profiles change among *Soja* seedling roots under neutral salt stress

**Figure 4:**
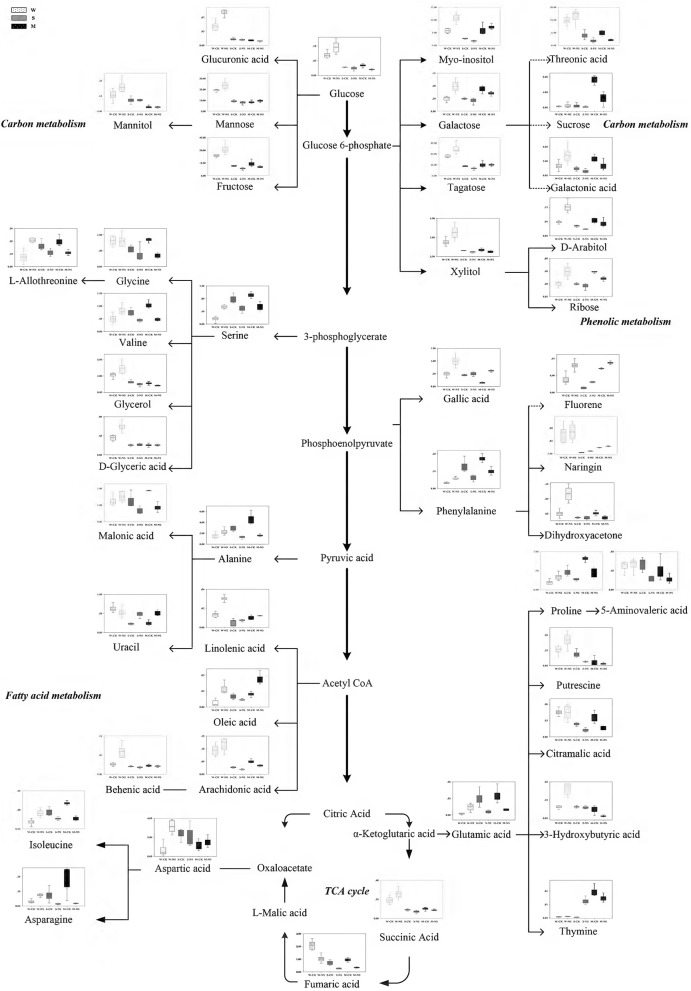
Changes in metabolites of the metabolic pathways in the roots of the three soybean genotypes seedlings varied with salt tolerance 14 days after the imposition of neutral salt stress. W, S, and M on the X-axis indicate wild soybean, semi-wild soybean, and cultivated soybean, respectively. The values on the Y-axis indicate the relative concentration of metabolites. CK, control treatment; NS, neutral salt stress.

According to the metabolomic analysis, the metabolites of *Soja* seedling roots were significantly changed under neutral salt stress (Table 2). We focused on 47 metabolites that affected the metabolic pathways (Figure 4). These included 11 metabolites related to amino acid metabolism, 7 related to fatty acid metabolism, 12 related to sugar alcohol metabolism, 5 related to carboxylic acids, 5 related to the TCA cycle, 5 related to antioxidants from secondary metabolism, and 2 kinds of nucleic acids. Amino acid, fatty acid, sugars alcohol, and carboxylic acid synthesis increased significantly in W under neutral salt stress; however, they were inhibited in S and M. The amino acids mainly included proline, isoleucine, serine, l-threonine, glutamic acid, valine, phenylalanine, and asparagines (*p* < 0.05). The saturated fatty acids were pentadecanoic acid, 24 alkyl acids and polyunsaturated fatty acids including 4 arachidonic acids, oleic acid, and linoleic acid, which are associated with fatty acid metabolism. Under neutral salt stress, the sugar and alcohol metabolism in the seedling roots of W increased significantly, including galactose, talose, d-arabinitol, inositol, dihydroxyacetone, and mannitol levels (*p* < 0.05). Nevertheless, most of the products of sugar alcohol metabolism declined in S and M. Additionally, ribose, d-arabinitol, xylitol, fructose, talose, and inositol were significantly reduced (*p* < 0.05) in S and glucose, sucrose, and dihydroxyacetone were reduced in M. Small molecule carboxylic acid metabolism was promoted in W and 3-hydroxy butyric acid and glucuronic acid increased significantly (*p* < 0.05), while in S and M, the opposite trend occurred, in which 5-aminovaleric acid was significantly decreased (*p* < 0.05). Under neutral salt stress, the TCA of *Soja* seedling roots was inhibited, but the inhibition in S and M was greater than W, and the fumaric acid, succinic acid, citric acid, and malonic acid metabolites were decreased. In W, the significant accumulations of metabolites related to ROCs, such as hydroxylamine and putrescine; however, the opposite trend occurred in S and M. Naringin and gallic acid were accumulated in all three genotypes, but the accumulation in W was more significant. In addition, uracil and thymidine, as regulators in W, S, and M, were increased.

**Table 2:** Relative concentration and fold changes of differential metabolites among *soja* seedling roots after 14 days of neutral-salt stress.

**Metabolite name**		**Relative concentration**						**Fold changes**${\text{log}}_{2}^{\left(NS/CK\right)}$		
		**W**		**S**		**M**				
		**CK**	**NS**	**CK**	**NS**	**CK**	**NS**	**W**	**S**	**M**
Amino acids	Proline	0.19 ± 0.03	0.34 ± 0.05	0.47 ± 0.06	0.28 ± 0.03	0.82 ± 0.04	0.44 ± 0.07	0.83	−0.76^*^	−0.87^**^
	Isoleucine	0.14 ± 0.04	0.32 ± 0.04	0.36 ± 0.04	0.22 ± 0.02	0.54 ± 0.01	0.22 ± 0.02	1.17^*^	−0.71^*^	−1.30^**^
	Serine	0.16 ± 0.03	0.54 ± 0.02	0.79 ± 0.07	0.48 ± 0.05	0.91 ± 0.02	0.54 ± 0.07	1.74^*^	−0.71^*^	−0.75^**^
	L-allothreonine	0.07 ± 0.03	0.21 ± 0.01	0.16 ± 0.01	0.11 ± 0.01	0.20 ± 0.02	0.11 ± 0.01	1.49^**^	−0.58	−0.86^**^
	Glutamic acid	0.00 ± 0.00	0.01 ± 0.00	0.03 ± 0.01	0.01 ± 0.00	0.03 ± 0.01	0.01 ± 0.00	2.55^**^	−2.32^*^	−1.87^*^
	Valine	0.50 ± 0.11	0.83 ± 0.11	0.75 ± 0.08	0.44 ± 0.03	1.04 ± 0.04	0.47 ± 0.03	0.74	−0.77^**^	−1.13^**^
	Alanine	1.56 ± 0.30	2.28 ± 0.33	2.90 ± 0.26	1.30 ± 0.13	4.64 ± 0.58	1.61 ± 0.11	0.55	−1.15^**^	−1.53^**^
	Phenylalanine	0.03 ± 0.00	0.06 ± 0.00	0.13 ± 0.01	0.06 ± 0.00	0.17 ± 0.01	0.10 ± 0.01	0.84^**^	−1.07^*^	−0.81^**^
	Asparagines	0.04 ± 0.00	0.07 ± 0.00	0.02 ± 0.00	0.01 ± 0.00	0.02 ± 0.00	0.02 ± 0.00	0.78^*^	−0.43	−0.20
	Glycine	0.81 ± 0.09	0.87 ± 0.06	0.55 ± 0.06	0.37 ± 0.07	0.74 ± 0.05	0.35 ± 0.02	0.10	−0.56	−1.08^**^
	Aspartic acid	0.64 ± 0.40	3.14 ± 0.39	2.35 ± 0.32	2.15 ± 0.59	0.86 ± 0.02	0.35 ± 0.02	2.29^**^	−0.13	−1.29
Fatty acids	Pentadecanoic acid	0.00 ± 0.00	0.02 ± 0.00	0.01 ± 0.00	0.01 ± 0.00	0.01 ± 0.00	0.01 ± 0.00	2.73^**^	−0.06	−0.20
	Lignoceric acid	0.03 ± 0.00	0.06 ± 0.01	0.02 ± 0.00	0.02 ± 0.00	0.02 ± 0.00	0.02 ± 0.00	1.18^*^	−0.17	−0.10
	Arachidonic acid	0.09 ± 0.01	0.14 ± 0.03	0.03 ± 0.00	0.02 ± 0.00	0.05 ± 0.00	0.03 ± 0.00	0.57	−0.50^**^	−0.57^**^
	Oleic acid	0.00 ± 0.00	0.02 ± 0.00	0.01 ± 0.00	0.01 ± 0.00	0.01 ± 0.00	0.01 ± 0.00	2.04^**^	−0.53	−0.22
	Linolenic acid	0.01 ± 0.00	0.03 ± 0.00	0.00 ± 0.00	0.01 ± 0.00	0.01 ± 0.00	0.01 ± 0.00	1.20^**^	0.61	0.26
	Glycerol	3.15 ± 0.27	4.39 ± 0.65	1.89 ± 0.15	1.45 ± 0.15	1.66 ± 0.15	1.22 ± 0.06	0.48	−0.38	−0.44^*^
	D-glyceric acid	0.36 ± 0.02	0.59 ± 0.03	0.20 ± 0.01	0.21 ± 0.03	0.20 ± 0.02	0.21 ± 0.01	0.71^*^	0.06	0.02
Carbohydrates and polyols	Glucose	0.07 ± 0.01	0.10 ± 0.01	0.03 ± 0.00	0.02 ± 0.00	0.03 ± 0.00	0.02 ± 0.00	0.46	−0.26	−0.75^*^
	Sucrose	0.28 ± 0.09	0.50 ± 0.33	0.42 ± 0.32	0.15 ± 0.10	7.21 ± 0.57	2.33 ± 0.87	0.82	−1.44	−1.63^**^
	Fructose	23.60 ± 0.45	31.22 ± 1.77	11.56 ± 0.46	8.48 ± 0.69	14.25 ± 1.87	10.38 ± 0.74	0.40	−0.45^**^	−0.46
	Ribose	7.28 ± 0.69	9.59 ± 1.09	2.75 ± 0.09	1.72 ± 1.46	4.36 ± 0.45	2.93 ± 0.05	0.40	−0.91^**^	−0.67^*^
	Galactose	0.20 ± 0.01	0.39 ± 0.03	0.20 ± 0.01	0.17 ± 0.02	0.35 ± 0.03	0.28 ± 0.02	0.98^*^	−0.24	−0.31
	Tagatose	18.06 ± 0.69	24.76 ± 2.61	8.76 ± 0.19	6.72 ± 0.57	10.10 ± 1.40	10.04 ± 0.69	0.46^*^	−0.38^*^	−0.01
	D-arabitol	0.06 ± 0.00	0.12 ± 0.01	0.05 ± 0.00	0.03 ± 0.00	0.06 ± 0.01	0.04 ± 0.01	1.09^**^	−0.54^**^	−0.60
	Xylitol	2.27 ± 0.33	3.81 ± 0.64	0.94 ± 0.02	0.72 ± 0.06	1.05 ± 0.13	0.78 ± 0.10	0.75	−0.39^*^	−0.43
	Myo-inositol	5.74 ± 0.54	10.81 ± 1.07	2.71 ± 0.14	1.70 ± 0.18	5.92 ± 1.11	7.26 ± 0.52	0.91	−0.67^**^	0.29
	Dihydroxyacetone	0.05 ± 0.01	0.11 ± 0.01	0.03 ± 0.00	0.03 ± 0.00	0.05 ± 0.00	0.03 ± 0.00	1.23^*^	−0.03	−0.49^*^
	Mannose	19.20 ± 0.63	24.17 ± 2.51	9.35 ± 0.27	7.92 ± 0.00	8.40 ± 0.71	9.33 ± 0.50	0.33	−0.24	0.15
	Mannitol	0.11 ± 0.02	0.16 ± 0.00	0.07 ± 0.01	0.07 ± 0.02	0.03 ± 0.00	0.03 ± 0.00	0.60	0.02	−0.06
TCA	Fumaric acid	2.10 ± 0.21	1.03 ± 0.17	0.71 ± 0.10	0.27 ± 0.03	0.95 ± 0.08	0.35 ± 0.03	−1.02^**^	−1.41^**^	−1.44^**^
	Succinic acid	0.19 ± 0.02	0.26 ± 0.02	0.09 ± 0.00	0.07 ± 0.00	0.10 ± 0.01	0.09 ± 0.00	0.43	−0.38	−0.19
	Citric acid	7.58 ± 1.00	6.89 ± 1.84	5.37 ± 1.19	2.26 ± 0.65	2.86 ± 0.41	1.18 ± 0.13	−0.14	−1.25	−1.27^*^
	Citramalic acid	0.05 ± 0.00	0.04 ± 0.01	0.02 ± 0.00	0.01 ± 0.00	0.04 ± 0.01	0.02 ± 0.00	−0.03	−0.92^**^	−1.14^*^
	Malonic acid	1.25 ± 0.18	1.54 ± 0.18	1.26 ± 0.22	0.67 ± 0.11	1.86 ± 0.01	0.85 ± 0.07	0.30	−0.91	−1.12^*^
Carboxylic acid	3-Hydroxybutyric acid	0.15 ± 0.00	0.35 ± 0.01	0.06 ± 0.00	0.06 ± 0.00	0.14 ± 0.06	0.01 ± 0.05	1.24^**^	−0.10^**^	−3.69
	Threonic acid	12.03 ± 0.57	13.81 ± 2.02	4.75 ± 0.99	2.22 ± 0.55	5.80 ± 0.69	2.53 ± 0.21	0.20	−1.10	−1.20^**^
	Galactonic acid	1.99 ± 0.01	4.20 ± 0.01	1.40 ± 0.21	0.88 ± 0.01	3.50 ± 0.37	2.00 ± 0.01	1.07	−0.67	−0.81
	Glucuronic acid	0.05 ± 0.01	0.08 ± 0.00	0.01 ± 0.00	0.01 ± 0.00	0.01 ± 0.00	0.01 ± 0.00	0.79^**^	−0.18	−0.22
	5-Aminovaleric acid	0.50 ± 0.07	0.53 ± 0.08	0.52 ± 0.07	0.23 ± 0.03	0.39 ± 0.12	0.22 ± 0.05	0.09	−1.18^*^	−0.84
ROC	Hydroxylamine	0.08 ± 0.00	0.11 ± 0.01	0.02 ± 0.00	0.01 ± 0.00	0.02 ± 0.01	0.02 ± 0.00	0.46^*^	−0.78	−0.13
	Putrescine	0.10 ± 0.00	0.17 ± 0.03	0.08 ± 0.01	0.03 ± 0.00	0.02 ± 0.01	0.01 ± 0.00	0.72	−1.56^**^	−0.90
	Naringin	0.02 ± 0.00	0.02 ± 0.00	0.00 ± 0.00	0.00 ± 0.00	0.01 ± 0.00	0.01 ± 0.00	0.17	1.26^**^	0.30
	Gallic acid	0.49 ± 0.05	0.99 ± 0.13	0.44 ± 0.02	0.50 ± 0.04	0.14 ± 0.01	0.61 ± 0.02	1.01^*^	0.16	2.10^**^
	Fluorene	0.00 ± 0.00	0.01 ± 0.00	0.00 ± 0.00	0.00 ± 0.00	0.02 ± 0.00	0.01 ± 0.00	1.08^**^	−1.31^**^	−0.70^**^
Nucleic acid	Uracil	0.52 ± 0.04	0.63 ± 0.03	0.23 ± 0.01	0.48 ± 0.02	0.25 ± 0.02	0.51 ± 0.02	0.27	1.06^**^	1.05^**^
	Thymine	0.00 ± 0.00	0.00 ± 0.00	0.00 ± 0.00	0.01 ± 0.00	0.01 ± 0.00	0.01 ± 0.00	0.47	4.77^**^	−0.42

*The relative contents of metabolites are the means of data from four biological replicates using GC−MS. The fold changes was calculated using the formula $\log_{\text{2}}^{\left(salt/control\right)}$. Values were presented as the mean ± standard deviation of four biological replicates. CK, control treatment; NS, neutral salt stress; AS, alkaline salt stress. W, wild soybean; S, semi-wild soybean; M, cultivated soybean*.

* *Significant difference at p < 0.05*.

** *Significant difference at p < 0.01*.

### Metabolic profile changes among *Soja* seedling roots under alkaline salt stress

**Figure 5:**
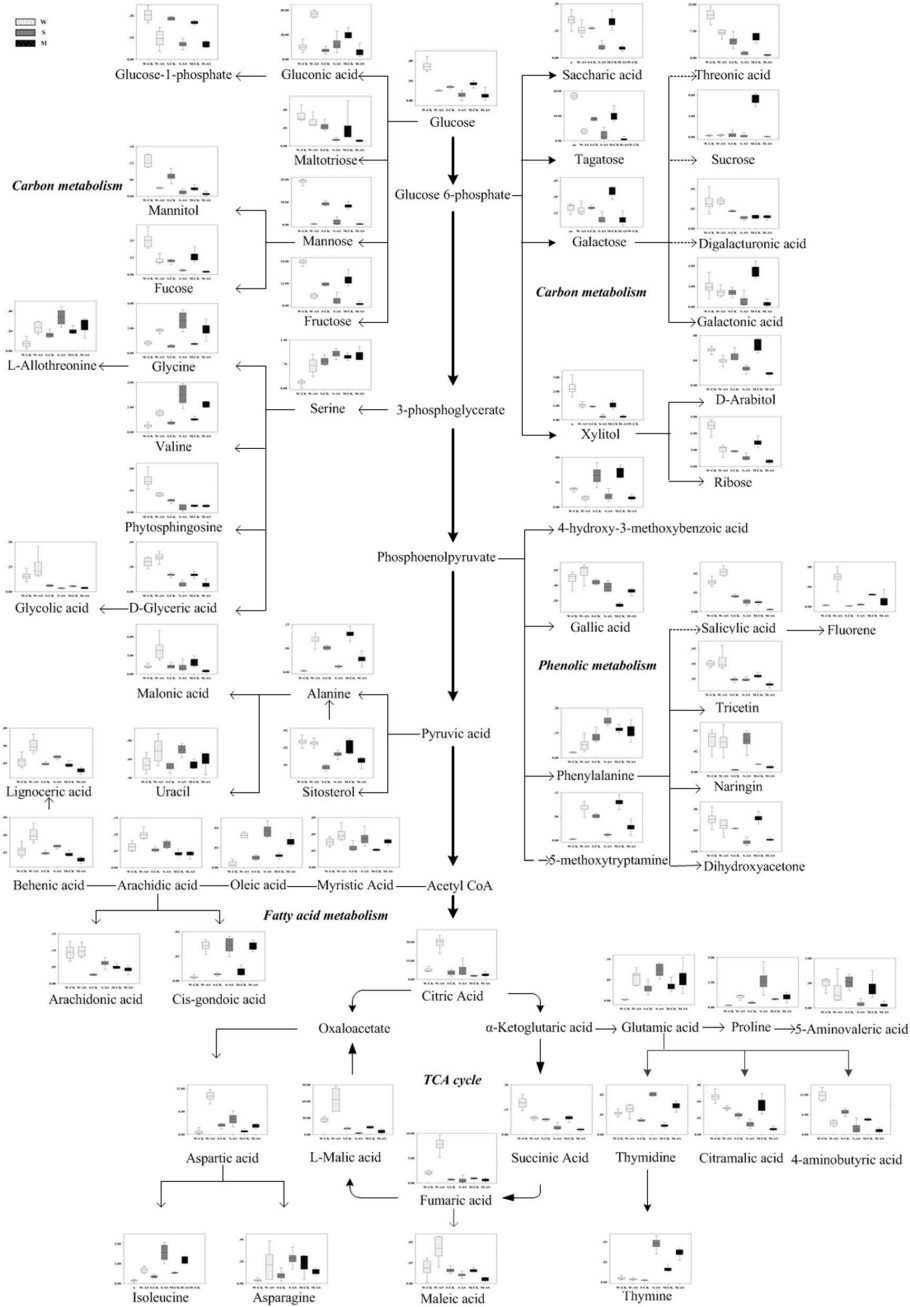
Changes in metabolites of the metabolic pathways in the roots of the three soybean genotypes seedlings varied with salt tolerance 14 days after the imposition of alkaline salt stress. W, S, and M on the X-axis indicate wild soybean, semi-wild soybean and cultivated soybean, respectively. The values on the Y-axis indicate the relative concentration of metabolites. CK, control treatment; AS, alkaline salt stress.

Based on the metabolomics analysis, the metabolites and the metabolic pathways of the *Soja* seedling roots under alkaline salt stress were changed more significantly than those under neutral salt stress (Table 3). There were 40 metabolites changes in W, 51 in S, and 43 in M under alkaline salt stress (*p* < 0.05). We focused on 62 common metabolic metabolites and metabolic pathways associated with these *Soja* seedling roots (Figure 5, Table 3). There were 11, 8, 8, 14, and 10 metabolites related to amino acid, fatty acid, TCA, sugar alcohol, and carboxylic acid metabolic pathways, respectively. There were also eight kinds of metabolites related to ROC and three kinds of nucleic acid metabolites. The amino acid metabolism of soybean under alkaline salt stress was enhanced and the change in W was significantly greater than in S and M. Proline, isoleucine, valine, and glycine accumulated significantly (*p* < 0.05). The fatty acid metabolism of arachidonic acid, behenic acid, oleic acid, trans-oleic acid, and 20 docosahexaenoic acids increased in soybean (*p* < 0.05) and, when compared with S and M, the W accumulation was more significant. Compared with under neutral salt stress, the TCA metabolic pathway was significantly altered. It was inhibited in S and M, with the degree of inhibition in S being greater than in M, while the intermediate products of W increased under alkaline salt stress. Under the alkaline salt stress, the sugar and alcohol metabolism in the three genotypes was significantly inhibited (*p* < 0.05), as were the carbohydrates, including talose, galactose, glucose, fucose, ribose, mannose, and fructose, and the alcohol metabolites, including mannitol, d-arabinitol, xylitol, and phytosphingosine. For these metabolites, the decreases were greater in S and M than in W. In addition, the trend for changes in the metabolism of carboxylic acids was similar to that of sugar alcohol metabolism, which mainly included 4-aminobutyric acid, threonic acid, 4-hydroxy-3-methoxybenzoic acid, and galacturonic acid. Changes in antioxidant metabolites were also more significant than under the neutral salt stress. In addition, five hydroxyl flavonoids, hydroxylamine, gallic acid, naringin 5-methoxytryptamine, sitosterol, and alicylic acid, were significantly accumulated (*p* < 0.05). Furthermore, three kinds of nucleic acids in *Soja* seedling roots showed an upward trend.

**Table 3:** Relative concentration and fold changed of differential metabolites among *soja* seedling roots after 14 days of alkaline salt stress.

**Metabolite name**		**Relative concentration**						**Fold changes**${\text{Log}}_{2}^{\left(AS/CK\right)}$		
		**W**		**S**		**M**				
		**CK**	**AS**	**CK**	**AS**	**CK**	**AS**	**W**	**S**	**M**
Amino acids	Proline	0.19 ± 0.03	1.07 ± 0.13	0.47 ± 0.06	2.70 ± 0.71	0.82 ± 0.04	1.02 ± 0.21	2.60^**^	2.53^*^	0.32
	Phenylalanine	0.03 ± 0.00	0.08 ± 0.01	0.13 ± 0.01	0.23 ± 0.01	0.17 ± 0.01	0.16 ± 0.01	1.30	0.84^*^	−0.08
	Glutamic acid	0.00 ± 0.00	0.05 ± 0.01	0.03 ± 0.01	0.07 ± 0.01	0.03 ± 0.01	0.05 ± 0.02	4.46^*^	1.29^*^	0.63
	Aspartic acid	0.64 ± 0.40	10.03 ± 0.81	2.35 ± 0.32	1.10 ± 0.90	0.86 ± 0.09	2.23 ± 0.15	3.97^**^	0.78	1.38^*^
	L-allothreonine	0.07 ± 0.03	0.23 ± 0.03	0.16 ± 0.01	0.34 ± 0.03	0.20 ± 0.02	0.25 ± 0.04	1.66^*^	1.05^*^	0.32
	Isoleucine	0.14 ± 0.04	0.69 ± 0.08	0.36 ± 0.04	1.56 ± 0.25	0.54 ± 0.01	1.16 ± 0.05	2.26^**^	2.13^**^	1.10^*^
	Valine	0.50 ± 0.11	1.54 ± 0.13	0.75 ± 0.08	3.00 ± 0.48	1.04 ± 0.04	2.19 ± 0.07	1.62^**^	1.99^**^	1.08^*^
	Glycine	0.81 ± 0.09	1.80 ± 0.10	0.55 ± 0.06	2.62 ± 0.43	0.74 ± 0.05	1.88 ± 0.37	1.15^**^	2.25^**^	1.35^*^
	Serine	0.16 ± 0.03	0.65 ± 0.07	0.79 ± 0.07	1.00 ± 0.07	0.91 ± 0.02	0.95 ± 0.05	2.00^*^	0.34	0.06
	Alanine	1.56 ± 0.30	5.15 ± 0.74	2.90 ± 0.26	6.65 ± 0.81	4.64 ± 0.58	5.91 ± 0.56	1.72^**^	1.20^**^	0.35
	Asparagine	0.03 ± 0.00	0.17 ± 0.04	0.07 ± 0.01	0.23 ± 0.02	0.19 ± 0.03	0.11 ± 0.01	2.49	1.63^**^	−0.79
Fatty acids	Arachidic acid	0.06 ± 0.01	0.10 ± 0.01	0.05 ± 0.00	0.07 ± 0.01	0.04 ± 0.00	0.04 ± 0.01	0.66^*^	0.38	−0.03
	Lignoceric acid	0.03 ± 0.00	0.04 ± 0.01	0.02 ± 0.00	0.03 ± 0.00	0.02 ± 0.00	0.02 ± 0.00	0.56	0.74^**^	−0.26
	Behenic acid	0.03 ± 0.00	0.06 ± 0.00	0.03 ± 0.00	0.04 ± 0.00	0.03 ± 0.00	0.02 ± 0.00	0.88	0.48^**^	−0.62^*^
	Arachidonic acid	0.09 ± 0.01	0.10 ± 0.01	0.03 ± 0.00	0.06 ± 0.01	0.05 ± 0.00	0.04 ± 0.01	0.06	1.22^**^	−0.26
	Oleic acid	0.00 ± 0.00	0.03 ± 0.00	0.01 ± 0.00	0.04 ± 0.00	0.01 ± 0.00	0.03 ± 0.00	3.17^**^	1.90^**^	1.15^**^
	Elaidic acid	0.09 ± 0.01	0.13 ± 0.04	0.05 ± 0.00	0.07 ± 0.01	0.06 ± 0.01	0.07 ± 0.01	0.46	0.69^*^	0.39
	Cis-gondoic acid	0.00 ± 0.00	0.03 ± 0.00	0.01 ± 0.00	0.03 ± 0.00	0.01 ± 0.00	0.03 ± 0.00	3.24^**^	2.40^**^	2.00^**^
	D-glyceric acid	0.36 ± 0.02	0.41 ± 0.03	0.20 ± 0.01	0.09 ± 0.01	0.20 ± 0.02	0.09 ± 0.03	0.19	−1.20^*^	−1.23^**^
TCA	Succinic acid	0.19 ± 0.02	0.10 ± 0.01	0.09 ± 0.00	0.04 ± 0.01	0.10 ± 0.01	0.03 ± 0.00	−0.94^**^	−1.06^**^	−1.74^**^
	Citramalic acid	0.05 ± 0.00	0.03 ± 0.00	0.02 ± 0.00	0.01 ± 0.00	0.04 ± 0.01	0.01 ± 0.00	−0.52^*^	−0.90^**^	−2.43^**^
	Glucose-1-phosphate	0.20 ± 0.02	0.10 ± 0.02	0.19 ± 0.00	0.07 ± 0.01	0.17 ± 0.00	0.07 ± 0.01	−1.09^*^	−1.42^**^	−1.29^**^
	Fumaric acid	2.10 ± 0.21	7.74 ± 0.93	0.71 ± 0.10	0.57 ± 0.35	0.95 ± 0.08	0.62 ± 0.18	1.88^**^	−0.32	−0.61
	L-malic acid	26.68 ± 1.30	63.42 ± 6.58	11.16 ± 1.27	2.49 ± 0.52	13.45 ± 1.34	5.95 ± 1.85	1.25^*^	−2.16^**^	−1.18^*^
	Citric acid	7.58 ± 1.00	29.38 ± 3.09	5.37 ± 1.19	7.40 ± 3.245	2.86 ± 0.41	3.85 ± 0.88	1.96^**^	0.46	0.43
	Malonic acid	1.25 ± 0.18	3.93 ± 1.07	1.26 ± 0.22	1.11 ± 0.42	1.86 ± 0.22	0.49 ± 0.05	1.65^*^	−0.18	−1.93^*^
	Maleic acid	0.02 ± 0.01	0.04 ± 0.01	0.02 ± 0.00	0.01 ± 0.00	0.02 ± 0.00	0.01 ± 0.00	1.18	−0.63	−1.51^**^
Carbohydrates and polyols	Mannitol	0.11 ± 0.02	0.03 ± 0.00	0.07 ± 0.01	0.02 ± 0.00	0.03 ± 0.00	0.01 ± 0.00	−1.83^*^	−2.27^**^	−1.80^*^
	D-Arabitol	0.06 ± 0.00	0.04 ± 0.00	0.05 ± 0.00	0.03 ± 0.00	0.06 ± 0.01	0.02 ± 0.00	−0.54^**^	−0.79^**^	−1.79^**^
	Dihydroxyacetone	0.05 ± 0.01	0.04 ± 0.01	0.03 ± 0.00	0.01 ± 0.00	0.05 ± 0.00	0.02 ± 0.00	−0.33	−1.27^**^	−1.48^**^
	Xylitol	2.27 ± 0.33	1.04 ± 0.09	0.94 ± 0.02	0.23 ± 0.05	1.05 ± 0.13	0.23 ± 0.05	−1.12^*^	−2.02^**^	−2.21^**^
	Phytosphingosine	0.06 ± 0.00	0.03 ± 0.00	0.02 ± 0.00	0.01 ± 0.00	0.01 ± 0.00	0.01 ± 0.00	−0.85^*^	−1.32^**^	−0.04
	Maltotriose	0.05 ± 0.00	0.05 ± 0.00	0.04 ± 0.00	0.01 ± 0.00	0.03 ± 0.01	0.01 ± 0.00	−0.16	−1.67^**^	−1.56^**^
	Tagatose	18.06 ± 0.69	3.83 ± 0.53	8.76 ± 0.19	2.31 ± 0.63	10.10 ± 1.40	0.61 ± 0.34	−2.24^**^	−1.92^**^	−4.04^**^
	Galactose	0.20 ± 0.01	0.18 ± 0.01	0.20 ± 0.01	0.09 ± 0.03	0.39 ± 0.02	0.09 ± 0.01	−0.18	−1.10^**^	−1.95^**^
	Glucose	0.07 ± 0.01	0.02 ± 0.00	0.03 ± 0.00	0.01 ± 0.00	0.03 ± 0.00	0.01 ± 0.00	−1.79^**^	−1.35^**^	−1.81^**^
	Fucose	0.24 ± 0.03	0.10 ± 0.02	0.10 ± 0.01	0.03 ± 0.00	0.13 ± 0.02	0.02 ± 0.00	−1.27^**^	−1.67^**^	−2.68^**^
	Ribose	7.28 ± 0.69	3.07 ± 0.49	2.75 ± 0.09	1.60 ± 0.30	4.36 ± 0.45	0.90 ± 0.19	−1.04^**^	−0.78^**^	−2.28^**^
	Mannose	19.20 ± 0.63	0.34 ± 0.11	9.35 ± 0.27	1.13 ± 0.44	8.40 ± 0.71	0.32 ± 0.20	−5.82^**^	−3.04^**^	−4.73^**^
	Fructose	23.60 ± 0.45	5.14 ± 0.37	11.56 ± 0.46	2.90 ± 1.47	14.25 ± 1.87	0.86 ± 0.47	−2.20^**^	−2.00^**^	−4.05^**^
	Sucrose	0.28 ± 0.09	0.33 ± 0.11	0.42 ± 0.32	0.20 ± 0.19	7.21 ± 0.57	0.05 ± 0.03	0.23	−1.03	−7.25^**^
Carboxylic acid	4-Aminobutyric acid	11.82 ± 0.96	3.47 ± 0.49	6.77 ± 0.49	2.00 ± 1.02	4.53 ± 0.22	1.21 ± 0.34	−1.77^**^	−1.76^**^	−1.90^**^
	5-Aminovaleric acid	0.50 ± 0.07	0.34 ± 0.15	0.52 ± 0.07	0.08 ± 0.04	0.39 ± 0.12	0.06 ± 0.03	−0.55	−2.66^**^	−2.72^*^
	Galactonic acid	1.99 ± 0.53	1.43 ± 0.26	1.40 ± 0.21	0.56 ± 0.35	3.50 ± 0.37	0.32 ± 0.17	−0.48	−1.34	−3.47^**^
	Saccharic acid	0.21 ± 0.02	0.16 ± 0.02	0.16 ± 0.00	0.06 ± 0.01	0.20 ± 0.02	0.05 ± 0.01	−0.44	−1.44^**^	−1.94^**^
	Threonic acid	12.03 ± 0.57	7.04 ± 0.31	4.75 ± 0.51	1.26 ± 0.13	5.80 ± 0.69	0.79 ± 0.13	−0.77^*^	−1.91^*^	−2.88^**^
	4-Hydroxy-3-methoxybenzoic acid	0.03 ± 0.00	0.01 ± 0.00	0.05 ± 0.01	0.02 ± 0.00	0.06 ± 0.01	0.01 ± 0.00	−0.99^**^	−1.50^*^	−1.90^**^
	Digalacturonic acid	0.03 ± 0.00	0.03 ± 0.00	0.02 ± 0.00	0.01 ± 0.00	0.01 ± 0.00	0.01 ± 0.00	0.05	−0.68^*^	−0.01
	Glycolic acid	0.16 ± 0.03	0.25 ± 0.07	0.06 ± 0.01	0.04 ± 0.00	0.06 ± 0.01	0.04 ± 0.00	0.64	−0.80^**^	−0.54^*^
	Gluconic acid	10.20 ± 1.17	36.88 ± 0.80	7.27 ± 0.72	12.53 ± 1.94	20.29 ± 1.05	6.10 ± 1.24	1.85^**^	0.78	−1.73^**^
	Myristic Acid	0.05 ± 0.00	0.06 ± 0.01	0.04 ± 0.00	0.05 ± 0.01	0.03 ± 0.00	0.05 ± 0.00	0.38	0.58	0.55^*^
Nucleic acid	Thymidine	0.01 ± 0.00	0.01 ± 0.00	0.01 ± 0.00	0.02 ± 0.00	0.00 ± 0.00	0.01 ± 0.00	0.23	1.49^**^	1.66^**^
	Thymine	0.00 ± 0.00	0.00 ± 0.00	0.00 ± 0.00	0.03 ± 0.00	0.01 ± 0.00	0.02 ± 0.00	−0.47	4.28^**^	1.15^**^
	Uracil	0.52 ± 0.04	0.73 ± 0.06	0.48 ± 0.02	0.75 ± 0.03	0.51 ± 0.02	0.59 ± 0.04	0.48	0.66^**^	0.20
ROC	Tricetin	0.02 ± 0.00	0.02 ± 0.00	0.01 ± 0.00	0.01 ± 0.00	0.01 ± 0.00	0.01 ± 0.00	0.11	0.00	−0.96^**^
	5-Methoxytryptamine	0.00 ± 0.00	0.18 ± 0.10	0.06 ± 0.00	0.01 ± 0.00	0.10 ± 0.00	0.03 ± 0.00	5.92^**^	−2.13^**^	−1.54^**^
	Sitosterol	0.02 ± 0.00	0.02 ± 0.00	0.01 ± 0.00	0.01 ± 0.00	0.02 ± 0.00	0.01 ± 0.00	−0.05	0.92^**^	−0.67
	Salicylic acid	0.02 ± 0.00	0.02 ± 0.00	0.01 ± 0.00	0.01 ± 0.00	0.00 ± 0.00	0.00 ± 0.00	0.42^*^	−0.63^*^	−2.09^**^
	Naringin	0.02 ± 0.00	0.02 ± 0.00	0.00 ± 0.00	0.02 ± 0.00	0.01 ± 0.00	0.00 ± 0.00	−0.07	3.93^**^	−0.64^**^
	Hydroxylamine	0.08 ± 0.00	0.05 ± 0.00	0.02 ± 0.00	0.02 ± 0.01	0.02 ± 0.01	0.01 ± 0.00	−0.80^**^	0.13	−0.71
	Gallic acid	0.49 ± 0.05	0.58 ± 0.06	0.44 ± 0.02	0.38 ± 0.04	0.14 ± 0.01	0.33 ± 0.02	0.24	−0.21	1.19^**^
	Fluorene	0.00 ± 0.00	0.06 ± 0.00	0.00 ± 0.00	0.00 ± 0.00	0.02 ± 0.00	0.01 ± 0.01	4.29^**^	1.58^*^	−1.15

*The relative contents of metabolites are the means of data from four biological replicates using GC–MS. The fold changes was calculated using the formula ${\text{log}}_{2}^{\left(salt/control\right)}$. Values were presented as the mean ± standard deviation of four biological replicates. CK, control treatment; NS, neutral salt stress; AS, alkaline salt stress. W, wild soybean; S, semi-wild soybean; M, cultivated soybean*.

* *Significant difference at p < 0.05*.

** *Significant difference at p < 0.01*.

## Discussion

Plant root systems are important for maintaining life activities by absorbing water and nutrients. They are closely related to the growth and yield of crops (Fan et al., 2013). Under salt stress, the sensitivities of plant organs are different, and the root system is very sensitive because it contacts the soil directly. Under salt stress, most plants suffer serious damage, and their growth and physiological metabolism are significantly inhibited (Munns, 2010). The growth and biomass accumulation among *Soja* were significantly inhibited. This demonstrated that the salt tolerance decreased regularly from wild to semi-wild to cultivated soybean which was consistent with our previous report (Zhang et al., 2016).

Based on metabolomics, the metabolites and their associated with metabolic pathways examined in this experiment changed among *Soja* seedling roots under neutral salt stress. When plants are subjected to salt stress, they can resist or reduce the damage caused by the accumulation of small molecule osmotic adjustment substances (Zhao and Zhang, 2013). Amino acid metabolism is enhanced and the small molecule amino acids accumulate, contributing to the enhanced salt tolerance ability by improving osmotic adjustments and maintaining cell membrane stability (Widodo et al., 2009). Our experimental results indicated that amino acid metabolism was significantly enhanced in wild soybean under neutral salt stress, leading to the significant accumulation of proline, glutamate, aspartate, isoleucine, glycine, alanine, and phenylalanine, whereas in semi-wild and cultivated soybean the result was opposite. Proline has the ability to resist salt stress, and the accumulation of glutamic acid is more helpful to proline synthesis and increases the proline content (Kumar et al., 2010). The synthesis of alanine is beneficial to reduce the sodium to potassium ratio in the plant, and the accumulation of isoleucine and phenylalanine may enhance glycolysis to alleviate salt stress. Additionally, these amino acids can increase the functions of scavenging free oxygen radicals, in addition to regulating osmotic pressure and alleviating salt stress (Wu et al., 2013a). Tagatose, d-arabitol, galactose significantly accumulated in wild soybean, revealing that sugar alcohol metabolism was enhanced. Mannitol and inositol are very important osmotic adjustment substances when plants are subjected to salt stress, and they also accumulate under these conditions (Conde et al., 2015). Thus, improving sugar and alcohol metabolism is an important way to alleviate the salt stress of wild soybean. The accumulation of organic acids such as 3-hydroxybutyric acid, galacturonic acid, and glycuronic acid was closely related to salt stress. Liu reported that fatty acids and their derivatives are also involved in plant resistance to abiotic stress, in addition to their roles in storing energy (Liu et al., 2014). 3-Hydroxybutyric acid plays an important role in improving membrane stability (Renault et al., 2010). Furthermore, 15 alkyl, oleic, and linoleic acids significantly accumulated in wild soybean as substances involved in the resistance of neutral salt stress. Under neutral salt stress, the TCA cycle was significantly inhibited in semi-wild and cultivated soybeans compared with in wild soybean, demonstrating that increased production capacity is not the main mechanism of salt resistance which was great different with leaves (Zhang et al., 2016). Under neutral salt stress, the secondary metabolism of antioxidants was enhanced in wild soybean, with significantly accumulated naringin, gallic acid, hydroxylamine, and putrescine. Naringin is aflavonoid, and its accumulation can enhance the resistance of plants (Tattini et al., 2004). Gallic acid is a polyphenol with strong antioxidant and anti-free radical effects (Zhang et al., 2011). A large amount of hydroxylamine and putrescine were accumulated in wild soybean under neutral salt stress, which can reduce the damage caused by salt stress by eliminating excess ROS (Conde et al., 2015). Additionally, hydroxylamine is an intermediate product of plants using nitrate ions, and its accumulation indicates that wild soybean was able to use more ${\text{NO}}_{3}^{-}$, which indicates that the nitrogen metabolism of wild soybean increased. During artificial breeding, the nitrogen fixation ability of cultivated soybean was greater, but the wild soybean improved the utilization of ${\text{NO}}_{3}^{-}$.

Amino acid metabolism was enhanced, while sugar alcohol metabolism and carboxylic acid metabolism were inhibited in the three genotypes' seedling roots in response to alkaline salt stress. Fatty acid metabolism in wild soybean and semi-wild soybean enhanced under alkaline salt stress; and decreased in cultivated soybean. Additionally, the secondary metabolism of antioxidants and the TCA cycle were enhanced in wild soybean; however, these were weakened in semi-wild and cultivated soybeans. At the same time, amino acids and fatty acid levels, the secondary metabolism of antioxidants and the TCA cycle were regularly changed in the seedling roots of wild semi-wild and cultivated soybean. The accumulation of amino acid in wild soybean was significantly greater than in semi-wild and cultivated soybeans, indicating that wild soybean can improve the resistance to alkaline salt stress by regulating the metabolism of amino acid. Fatty acid metabolism was enhanced in wild and semi-wild soybeans, indicating that it was closely related to the *Soja* response to alkaline salt stress. In addition, comparing with under neutral salt stress the metabolites were more abundant; in particular, arachidonic and eicosapentaenoic acids were significantly increased. The TCA cycle is an important energy-producing process in plants, and it plays an important role in resisting adverse environmental conditions (Dhiman et al., 2012). Intermediate metabolites including fumaric acid, malic acid, citric acid, and malonic acid were accumulated in the TCA cycle, revealing that the cycle was significantly enhanced under alkaline salt stress. Wild soybean generated more energy to resist alkaline salt stress, but the levels were decreased in semi-wild and cultivated soybeans' seedling roots. Citric acid and malicacid are intermediate products in the TCA cycle but also scavenged active oxygen species. Thus, TCA can enhance the salt tolerance of wild soybean by increasing the energy capacity and the levels of intermediate products under alkaline salt stress, which was similar to *P. pratensis* (Hu et al., 2014). The trend of secondary metabolism changes of antioxidants was similar under both neutral and alkaline salt stresses. However, there are significant differences in their species and numbers; the most obvious is the enhancement of phenolic metabolism, especially those of quercetin, sitosterol, and salicylic acid. Quercetin is a flavonoid, it is a secondary metabolite of plant synthesis, and has the protective effect of aiding in the adaptation to stress (Shi et al., 2013). The accumulation of sitosterol in wild and semi-wild soybeans was significant under alkaline salt stress, having an antioxidant effect, while in plants sitosterol plays a role in stabilizing the cell membrane (Azooz, 2009). Salicylic acid is significantly accumulated in wild soybean, has an important role in plant salt tolerance, and it can induce or enhance the antioxidant system to remove excessive ROS in plants, reducing the degree of cell membrane lipid peroxidation, improving cell metabolic activities and alleviating the inhibition of salt stress in plants (Zhao and Zhang, 2013). This suggests that it has a close relationship with the reduction in salt-induced injury.

The determination of biomass showed that the salt tolerance decreased regularly from wild to semi-wild to cultivated soybean. Meanwhile, the small molecular metabolites in the metabolism were very significantly different between roots and leaves under salt stress. Roots of wild soybean seedling roots could regulate nitrogen and carbon metabolism, as well as the secondary metabolism of antioxidants to resist natural salt stress, and regulate nitrogen metabolism, the secondary metabolism of antioxidants, and the TCA cycle to adapt to alkaline salt stress. The data analysis showed that the changes in semi-wild soybean under both types of salt stress were more similar to those of cultivated soybean than those of wild soybean. The analysis also confirmed that semi-wild soybean is an intermediate type of *Soja*. During the long domestication and continuous artificial breeding processes, the physiology and metabolism of cultivated soybean adapted to human needs. Consistent with previous studies, the metabolic processes in the cultivated soybean are more sensitive to adverse environmental conditions, especially salinity, and protein metabolism and fatty acid metabolism in soybean are more vulnerable to salt stress (Hyun et al., 2015).

## Conclusion

The salt tolerance of wild soybean was the greatest, while that of cultivated soybean was the weakest. Wild soybean's salt tolerance mechanism is mainly dependent on increases in nitrogen metabolism and antioxidant metabolism of secondary metabolites. In addition, carbon metabolism is also important in resisting neutral salt, and the resistance to alkaline salt stress is also dependent on the enhancement of the TCA cycle. Semi-wild and cultivated soybeans' metabolism of carbon and nitrogen and TCA and ROC, as well as other physiological metabolisms, are very sensitive, especially in cultivated soybean. Our study revealed that the physiological metabolisms of carbon and nitrogen are key factors of salt tolerance among *Soja*. This study provides new insights into salt resistance in soybean and presents a quantitative parameter system for the cultivation of saline alkaline resistant soybean.

## Author contributions

ML, RG, and LS designed the research. ML and RG performed the research. ML, RG, and YJ analyzed the data, and ML, RG, YJ, XJ, HZ, and LS wrote the paper. All authors reviewed the manuscript.

### Conflict of interest statement

The authors declare that the research was conducted in the absence of any commercial or financial relationships that could be construed as a potential conflict of interest. The reviewer TI and handling Editor declared their shared affiliation, and the handling Editor states that the process nevertheless met the standards of a fair and objective review.
