# Supplementary material for: Comparison of Salt Tolerance in Soja Based on Metabolomics of Seedling Roots
Source: Front Plant Sci. 2017 Jun 23;8:1101. doi: 10.3389/fpls.2017.01101 (PMC5481370; doi:10.3389/fpls.2017.01101)
Supplement: Supplementary file 1 [file Table1.DOCX]

***Supplementary Material***

**Comparison of salt tolerance in *Soja* based on metabolomics of** **seedling roots**

**Mingxia Li^1†^, Rui Guo^2†^, Yang Jiao^1^, Xiaofei Jin^3^, Haiyan Zhang****^1^** **and Lianxuan Shi^1*^**

***Correspondence:** Dr. Lianxuan Shi**:** Email: [lianxuanshi@nenu.edu.cn](mailto:lianxuanshi@nenu.edu.cn)

# Supplementary TABLES

**Supplementary** **TABLE** **1 | The contribution of metabolites** **among *soja* seedling roots to the first principal component (PC1) and the second principal component (PC2).**

| metabolite name | PC1 | PC2 |
| --- | --- | --- |
| 4-Aminobutyric acid | 0.06 | 0.00 |
| Stearic acid | 0.04 | -0.01 |
| Fructose | 0.06 | -0.03 |
| Myo-inositol | 0.06 | -0.04 |
| Palmitic acid | 0.04 | 0.00 |
| Glycerol | 0.05 | -0.02 |
| Gluconic acid | 0.03 | -0.09 |
| Succinic acid | 0.05 | -0.04 |
| Tagatose | 0.06 | -0.03 |
| Fumaric acid | 0.05 | -0.07 |
| L-Allothreonine | -0.02 | -0.01 |
| D-Glyceric acid | 0.06 | -0.03 |
| Myo-inositol | 0.03 | -0.09 |
| Valine | -0.02 | -0.02 |
| Mannose | 0.05 | -0.02 |
| Citric acid | 0.04 | 0.00 |
| Ribose | 0.05 | -0.07 |
| Serine | -0.03 | 0.00 |
| Galactose | 0.05 | -0.01 |
| Oxoproline | 0.02 | -0.04 |
| Aspartic acid | 0.00 | 0.04 |
| Isoleucine | -0.03 | -0.01 |
| Uracil | 0.01 | -0.03 |
| Sucrose | 0.01 | -0.15 |
| Threonic acid | 0.07 | -0.05 |
| Glycine | 0.00 | -0.01 |
| Galactonic acid | 0.04 | -0.07 |
| Daidzein | 0.08 | -0.03 |
| Galactonic acid | 0.03 | 0.00 |
| 5-Aminovaleric acid | 0.05 | -0.02 |
| Xylitol | 0.06 | -0.04 |
| Lactic acid | 0.02 | -0.07 |
| Lyxose | 0.04 | -0.04 |
| 6-Deoxy-D-glucose | 0.04 | -0.03 |
| 1-Monopalmitin | 0.06 | -0.04 |
| Malonic acid | 0.03 | -0.04 |
| Cellobiose | 0.07 | -0.03 |
| Mannitol | 0.07 | 0.01 |
| Linoleic acid | 0.04 | -0.04 |
| Galactinol | 0.06 | -0.05 |
| D-Altrose | 0.07 | 0.01 |
| Fucose | 0.02 | -0.04 |
| Glucose-1-phosphate | 0.03 | -0.02 |
| Galactonic acid | 0.02 | -0.07 |
| Oleic acid | 0.01 | -0.03 |
| Galactonic acid | 0.06 | -0.04 |
| Heptadecanoic acid | 0.01 | -0.03 |
| Behenic acid | 0.04 | 0.00 |
| Threitol | 0.05 | -0.04 |
| Myristic Acid | 0.02 | -0.02 |
| Glutaric Acid | 0.05 | -0.01 |
| Lignoceric acid | 0.03 | -0.02 |
| Tricetin | 0.01 | -0.03 |
| Beta-Mannosylglycerate | 0.05 | 0.02 |
| Pelargonic acid | 0.04 | -0.04 |
| Arachidic acid | 0.04 | -0.02 |
| 2-Hydroxypyridine | 0.07 | 0.01 |
| Threonine | 0.04 | -0.02 |
| Arachidonic acid | 0.04 | -0.07 |
| L-Malic acid | 0.05 | -0.06 |
| Creatine degr | 0.02 | -0.02 |
| 1,2,4-Benzenetriol | 0.01 | -0.01 |
| 1,5-Anhydroglucitol | 0.06 | -0.06 |
| 3-Hydroxybutyric acid | 0.04 | -0.01 |
| Linoleic acid methyl ester | 0.04 | -0.01 |
| Linoleic acid methyl ester | 0.05 | -0.02 |
| Dihydroxyacetone | 0.03 | 0.00 |
| Phytosphingosine | 0.05 | 0.00 |
| Dihydroxyacetone | 0.01 | 0.01 |
| Linoleic acid methyl ester | 0.04 | -0.02 |
| Phytosphingosine | 0.00 | 0.02 |
| 2-Deoxyerythritol | 0.08 | 0.01 |
| Methyl Palmitoleate | 0.04 | -0.02 |
| Methyl Palmitoleate | 0.04 | -0.02 |
| Linoleic acid methyl ester | 0.05 | -0.04 |
| Fluorene | 0.04 | -0.09 |
| 1-Hexadecanol | 0.05 | -0.01 |
| Prostaglandin | 0.06 | -0.04 |
| Methyl Palmitoleate | 0.05 | -0.02 |
| Fucose | 0.08 | -0.01 |
| Hydroxylamine | 0.06 | -0.04 |
| Creatine degr | 0.04 | -0.01 |
| Tetracosane | 0.04 | -0.03 |
| Adipamide | -0.06 | 0.00 |
| Gallic acid | 0.03 | 0.04 |
| L-Malic acid | 0.06 | -0.07 |
| Alanine | -0.02 | -0.04 |
| Proline | -0.03 | -0.02 |
| 3-Hydroxy-3-methylglutaric acid | 0.04 | -0.05 |
| Succinic acid | 0.06 | -0.04 |
| Glycolic acid | 0.06 | -0.04 |
| Saccharic acid | 0.02 | -0.03 |
| Maltotriose | 0.07 | 0.03 |
| Methyl Phosphate | 0.05 | 0.00 |
| Threitol | -0.02 | 0.03 |
| Alanine | 0.00 | -0.06 |
| Tricetin | 0.05 | -0.05 |
| 3-Hydroxybutyric acid | 0.03 | -0.02 |
| Ethanolamine | 0.06 | 0.00 |
| 4-Hydroxy-3-methoxybenzoic acid; | 0.01 | -0.03 |
| Beta-Mannosylglycerate | 0.06 | -0.02 |
| Myo-inositol | 0.03 | -0.10 |
| Azelaic acid | 0.04 | -0.05 |
| 3-Cyanoalanine | 0.02 | -0.03 |
| Glycerol | 0.05 | -0.04 |
| Leucine | 0.04 | 0.04 |
| Beta-Alanine | 0.02 | -0.06 |
| Phenylalanine | -0.05 | -0.02 |
| Elaidic acid | 0.03 | -0.03 |
| Saccharic acid | 0.04 | -0.05 |
| Digitoxose | 0.06 | -0.05 |
| Conduritol bepoxide | 0.04 | -0.02 |
| Citramalic acid | 0.05 | -0.06 |
| Dihydroxyacetone | 0.04 | -0.04 |
| Gluconic lactone | 0.02 | -0.03 |
| Mannitol | 0.00 | -0.04 |
| N-Acetyl-D-galactosamine | 0.06 | -0.01 |
| Citric acid | 0.03 | -0.02 |
| 21-Hydroxypregnenolone | 0.05 | -0.03 |
| Pentadecanoic acid | -0.05 | 0.02 |
| 2-Deoxyerythritol | 0.06 | 0.00 |
| Lyxose | 0.03 | -0.04 |
| 22-Ketocholesterol | 0.07 | -0.03 |
| Putrescine | 0.07 | 0.03 |
| Linolenic acid | 0.03 | -0.04 |
| Beta-Mannosylglycerate | 0.03 | -0.06 |
| Myo-inositol | 0.04 | -0.07 |
| Threonic acid | 0.06 | -0.04 |
| Galactonic acid | 0.04 | -0.03 |
| 21-Hydroxypregnenolone | 0.01 | -0.02 |
| Mannose | 0.06 | -0.01 |
| Maleic acid | 0.03 | -0.08 |
| Glucose | 0.06 | -0.03 |
| Monostearin | 0.05 | -0.04 |
| Hydroxylamine | 0.00 | 0.01 |
| D-erythronolactone | 0.06 | 0.03 |
| Xylose | 0.04 | -0.03 |
| 4-Hydroxybutyrate | 0.08 | 0.03 |
| Glutamic acid | -0.05 | -0.01 |
| D-erythronolactone | 0.04 | -0.04 |
| Xylitol | 0.05 | -0.02 |
| Tagatose | -0.01 | -0.10 |
| Alpha-ketoisocaproic acid | 0.04 | 0.01 |
| Oleic acid | -0.01 | -0.02 |
| 2-Deoxyerythritol | 0.08 | -0.02 |
| Cellobiose | 0.07 | 0.00 |
| Sophorose | 0.04 | 0.03 |
| Galactinol | 0.02 | -0.09 |
| Beta-Glycerophosphoric acid | 0.02 | -0.07 |
| D-Arabitol | 0.04 | -0.06 |
| Asparagine | 0.06 | -0.03 |
| Gluconic lactone | -0.04 | 0.00 |
| Thymidine | 0.02 | -0.14 |
| Oxalic acid | 0.04 | -0.02 |
| D-erythro-sphingosine | 0.07 | -0.05 |
| 5-Aminovaleric acid | 0.07 | 0.01 |
| 1,2,4-Benzenetriol | -0.03 | 0.02 |
| Phytosphingosine | 0.05 | -0.04 |
| Azelaic acid | 0.04 | -0.01 |
| Glucose-1-phosphate | 0.02 | -0.03 |
| 2-Furoic Acid | 0.05 | -0.02 |
| Salicylic acid | 0.08 | 0.05 |
| 4-Aminobutyric acid | -0.11 | 0.06 |
| Digalacturonic acid | 0.11 | 0.20 |
| Cis-gondoic acid | -0.10 | 0.03 |
| D-Glucoheptose | -0.10 | 0.04 |
| 3-Hydroxybutyric acid | -0.03 | -0.13 |
| Gentiobiose | 0.04 | 0.14 |
| Maltotriose | -0.02 | -0.12 |
| Cycloleucine | 0.07 | 0.23 |
| 3-Hydroxy-3-methylglutaric acid | -0.09 | 0.03 |
| Galactonic acid | 0.03 | -0.08 |
| 5-Aminovaleric acid | 0.10 | 0.04 |
| Phytosphingosine | 0.12 | 0.19 |
| Fucose | 0.07 | -0.03 |
| Allo-inositol | 0.09 | 0.04 |
| Glycerol | -0.02 | 0.03 |
| Sitosterol | 0.02 | -0.22 |
| 2-Deoxyerythritol | 0.14 | 0.00 |
| Beta-Mannosylglycerate | 0.03 | -0.04 |
| 1-Hydroxyanthraquinone | 0.04 | -0.02 |
| Gluconic lactone | 0.05 | -0.04 |
| 2-Deoxyerythritol | 0.07 | 0.00 |
| Lactose | -0.11 | 0.04 |
| Iminodiacetic acid | 0.00 | -0.04 |
| Asparagine | -0.11 | 0.02 |
| 4-Hydroxybenzaldehyde | 0.05 | 0.01 |
| O-Hydroxyhippuric acid | -0.12 | 0.10 |
| Maltotriose | 0.12 | 0.04 |
| Trans-sinapinic acid | -0.10 | 0.20 |
| Tricetin | -0.03 | 0.05 |
| Galactose | -0.06 | 0.13 |
| Naringin | 0.10 | 0.19 |
| 2-Methylfumarate | 0.04 | 0.03 |
| Creatine degr | 0.15 | -0.06 |
| 5-Methoxytryptamine | -0.03 | 0.01 |
| Beta-Mannosylglycerate | 0.06 | -0.24 |
| Creatine degr | -0.14 | 0.06 |
| Gluconic acid | 0.14 | -0.06 |
| Maltotriose | -0.11 | 0.02 |
| Arachidic acid | -0.13 | -0.02 |
| Fluorene | -0.04 | -0.16 |
| Gluconic lactone | 0.15 | 0.01 |
| Leucrose | -0.06 | 0.16 |
| Galactonic acid | 0.03 | -0.07 |
| Neohesperidin | 0.13 | 0.01 |
| 2-Deoxyerythritol | 0.15 | -0.06 |
| Thymine | -0.12 | -0.02 |
| Terephthalic acid | 0.13 | 0.01 |
| 2,2-Dimethylsuccinic Acid | -0.13 | 0.09 |
| 5-Dihydrocortisol | 0.01 | -0.01 |
| Tricetin | 0.04 | 0.09 |
| D-Erythro-sphingosine | -0.04 | -0.04 |
| Alpha-ketoisocaproic acid | 0.05 | -0.02 |
| 1-Monopalmitin | -0.02 | 0.00 |
| 2-Furoic Acid | 0.02 | -0.17 |
| Gentiobiose | -0.11 | -0.17 |
| 2-Deoxyerythritol | 0.13 | -0.19 |
| Galactonic acid | -0.12 | -0.05 |
| N-Acetyl-D-galactosamine | -0.05 | -0.05 |
| Glucuronic acid | 0.14 | -0.06 |
| Gluconic lactone | 0.15 | -0.07 |
| Gluconic acid | -0.11 | -0.17 |
| D-Altrose | 0.15 | -0.06 |
| Digalacturonic acid | -0.11 | -0.19 |
| Maltotriose | -0.02 | -0.05 |
| 3-Hydroxy-3-methylglutaric acid | 0.14 | -0.06 |
| Itaconic acid | 0.15 | -0.07 |
| Pentadecanoic acid | -0.03 | 0.01 |
| Lyxonic acid, 1,4-lactone | -0.03 | 0.02 |
| Maltotriose | -0.09 | -0.08 |
| Naringin | -0.11 | -0.04 |
| Fluorene | 0.11 | -0.10 |
| Beta-Mannosylglycerate | -0.09 | -0.24 |
| Norleucine | -0.13 | -0.17 |
| Pentadecanoic acid | -0.05 | -0.10 |
| Diglycerol | -0.05 | -0.22 |
