# Supplementary material for: Comparison of Salt Tolerance in Soja Based on Metabolomics of Seedling Roots
Source: Front Plant Sci. 2017 Jun 23;8:1101. doi: 10.3389/fpls.2017.01101 (PMC5481370; doi:10.3389/fpls.2017.01101)
Supplement: Supplementary file 2 [file Table2.DOCX]

***Supplementary Material***

**Comparison of salt tolerance in *Soja* based on metabolomics of** **seedling roots**

**Mingxia Li^1†^, Rui Guo^2†^, Yang Jiao^1^,** **Xiaofei Jin^3^, Haiyan Zhang^1^ and Lianxuan Shi^1*^**

***Correspondence:** Dr. Lianxuan Shi**:** Email: [lianxuanshi@nenu.edu.cn](mailto:lianxuanshi@nenu.edu.cn)

# Supplementary TABLES

**Supplementary** **TABLE 2** **| Metabolite profiles changes** **among *soja* seedling roots under normal conditions.**

| metabolite name | log_2_^(S/W)^ | log_2_^(M/W)^ |
| --- | --- | --- |
| Proline | 1.31** | 2.11** |
| Phenylalanine | 2.12** | 2.50** |
| Glutamic acid | 3.63* | 3.63** |
| Aspartic acid | 1.88* | 0.43 |
| L-allothreonine | 1.19* | 1.51** |
| Isoleucine | 1.36** | 1.95** |
| Valine | 0.58 | 1.06** |
| Glycine | -0.56* | -0.13 |
| Serine | 2.30** | 2.51** |
| Alanine | 0.89* | 1.57** |
| Asparagine | 1.22* | 2.66 |
| Arachidic acid | -0.26 | -0.58 |
| Lignoceric acid | -0.58 | -0.58 |
| Behenic acid | 0.00 | 0.00 |
| Arachidonic acid | -1.58** | -0.85* |
| Oleic acid | 1.48 | 1.48* |
| Elaidic acid | -0.85** | -0.58* |
| Cis-gondoic acid | 1.72** | 1.72** |
| D-glyceric acid | -0.85* | -0.85 |
| Pentadecanoic acid | 1.72** | 1.72** |
| Linolenic acid | -0.58** | -0.58 * |
| Glycerol | -0.74** | -0.92** |
| Succinic acid | -1.08** | -0.93** |
| Citramalic acid | -1.32** | -0.32 |
| Glucose-1-phosphate | -0.07 | -0.23 |
| Fumaric acid | -1.56** | -1.14** |
| L-malic acid | -1.26 | -0.99** |
| Citric acid | -0.50 | -1.41 ** |
| Malonic acid | 0.01 | 0.57 |
| Maleic acid | 0.00 | 0.00 |
| Mannitol | -0.65* | -1.87 * |
| D-arabitol | -0.26** | 0.00 |
| Dihydroxyacetone | -0.74 | 0.00 |
| Xylitol | -1.27** | -1.11 * |
| Phytosphingosine | -1.58 | -2.58 ** |
| Maltotriose | -0.32** | -0.74 ** |
| Tagatose | -1.04** | -0.84 ** |
| Galactose | 0.00 | 0.96 * |
| Glucose | -1.22** | -1.22** |
| Fucose | -1.26** | -0.88 * |
| Ribose | -1.40** | -0.74 * |
| Mannose | -1.04** | -1.19** |
| Fructose | -1.03** | -0.73 ** |
| Sucrose | 0.58 | 4.69 ** |
| Myo-inositol | -1.08** | 0.04 |
| 4-aminobutyric acid | -0.80** | -1.38 ** |
| 5-aminovaleric acid | 0.06 | -0.36 |
| Galactonic acid | -0.51 | 0.81 |
| Saccharic acid | -0.39 | -0.07 |
| Threonic acid | -1.34** | -1.05 ** |
| 4-hydroxy-3-methoxybenzoic acid | 0.74* | 1.00 ** |
| Digalacturonic acid | -0.58 | -1.58 * |
| Glycolic acid | -1.42* | -1.42 * |
| Gluconic acid | -0.49 | 0.99 * |
| Myristic acid | -0.32* | -0.74* |
| 3-hydroxybutyric acid | -1.32 | -0.10 |
| Glucuronic acid | -2.32** | -2.32** |
| Thymidine | 0.00 | -1.14 ** |
| Thymine | -0.68 | 4.76 ** |
| Uracil | -0.12 | -0.03 |
| Tricetin | -1.00** | -1.00 ** |
| 5-methoxytryptamine | 4.31** | 5.04 ** |
| Sitosterol | -1.00** | 0.00 |
| Salicylic acid | -1.00** | -2.08 ** |
| Naringin | -4.28 | -1.00 ** |
| Hydroxylamine | -2.00** | -2.00 ** |
| Gallic acid | -0.16 | -1.81 ** |
| Fluorene | 1.03** | 4.78 ** |
| Putrescine | -0.32** | -2.32 * |
