# Supplementary material for: Comparison of Salt Tolerance in Soja Based on Metabolomics of Seedling Roots
Source: Front Plant Sci. 2017 Jun 23;8:1101. doi: 10.3389/fpls.2017.01101 (PMC5481370; doi:10.3389/fpls.2017.01101)
Supplement: Supplementary file 3 [file Table3.DOCX]

***Supplementary Material***

**Comparison of salt tolerance in *Soja* based on metabolomics of** **seedling roots**

**Mingxia Li^1†^, Rui Guo^2†^, Yang Jiao^1^, Xiaofei Jin^3^, Haiyan Zhang^1^ and** **Lianxuan Shi^1*^**

***Correspondence:** Dr. Lianxuan Shi**:** Email: [lianxuanshi@nenu.edu.cn](mailto:lianxuanshi@nenu.edu.cn)

# Supplementary TABLES

**Supplementary** **TABLE 3 |** **Differences of metabolites profiles among *soja* seedling roots under neutral salt stress and alkaline salt stress.**

| metabolite name | log_2_^(treatment/control)^ | | | | | |
| --- | --- | --- | --- | --- | --- | --- |
|  | W(NS/CK) | W(AS/CK) | S(NS/CK) | S(AS/CK) | M(NS/CK) | M(AS/CK) |
| Proline | 0.84 | 2.49** | -0.75* | 2.52* | -0.90** | 0.31 |
| Phenylalanine | 1.00** | 1.42 | -1.12* | 0.82* | -0.77** | -0.09 |
| Glutamic acid | 2.04** | 4.36* | -1.58* | 1.22* | -1.58* | 0.74 |
| Aspartic acid | 2.29** | 3.97** | -0.13 | -1.10 | -1.30 | 1.37 |
| L-allothreonine | 1.58** | 1.72* | -0.54 | 1.09* | -0.86** | 0.32 |
| Isoleucine | 1.19* | 2.30** | -0.71* | 2.12** | -1.30** | 1.10* |
| Valine | 0.73 | 1.62** | -0.77** | 2.00** | -1.15** | 1.07* |
| Glycine | 0.10 | 1.15** | -0.57 | 2.25** | -1.08** | 1.35* |
| Serine | 1.75* | 2.02* | -0.72* | 0.34 | -0.75** | 0.06 |
| Alanine | 0.55 | 1.72** | -1.16** | 1.20** | -1.53** | 0.35 |
| Asparagine | 1.22 | 2.50 | -2.81 | 1.72** | -3.25 | -0.79 |
| Arachidic acid | 1.18* | 0.74* | -0.62** | 0.49 | -0.05 | 0.06 |
| Lignoceric acid | 1.00* | 0.42 | 0.00** | 0.58** | 0.08 | -0.09 |
| Behenic acid | 1.57 | 1.00 | -0.11 | 0.42** | -0.51 | -0.58* |
| Arachidonic acid | 0.64 | 0.15 | -0.58** | 1.00** | -0.74** | -0.32 |
| Oleic acid | 2.48* | 3.06** | -0.54 | 2.00** | 0.04 | 1.58** |
| Elaidic acid | 0.84 | 0.53 | -0.10 | 0.49* | 0.02 | 0.22 |
| Cis-gondoic acid | -6.59 | 3.31** | -1.12 | 1.58** | -0.53 | 1.58** |
| D-glyceric acid | 0.71* | 0.19 | 0.07 | -1.15* | 0.07 | -1.15** |
| Pentadecanoic acid | 2.72** | 2.30** | -0.44 | 0.55** | -0.20 | -0.29 |
| Linolenic acid | -0.14** | -1.50 | -1.00 | -0.66 | -1.00 | -1.36 |
| Glycerol | 0.48 | -0.82 | -0.38 | -0.89** | -0.44* | -1.10** |
| Succinic acid | 0.45 | -0.93** | -0.36 | -1.17** | -0.15 | -1.74** |
| Citramalic acid | -0.32 | -0.74* | -1.00** | -1.00** | -1.00* | -2.00** |
| Glucose-1-phosphate | 0.93* | -1.00* | -0.43** | -1.44** | 0.16* | -1.28** |
| Fumaric acid | -1.03** | 1.88** | -1.39** | -0.32 | -1.44** | -0.62 |
| L-malic acid | -0.95** | 1.25* | -1.42** | -2.16** | -1.69** | -1.18* |
| Citric acid | -0.14 | 1.95** | -1.25 | 0.46 | -1.28* | 0.43 |
| Malonic acid | 0.30 | 1.65* | -0.91 | -0.18 | -1.13* | -1.92* |
| Maleic acid | 0.28 | 1.00 | -2.24* | -1.00 | -0.96* | -1.00** |
| Mannitol | 0.54 | -1.87* | 0.09 | -1.81** | -0.16 | -1.58* |
| D-arabitol | 1.00** | -0.58** | -0.70** | -0.81** | -0.58 | -1.58** |
| Dihydroxyacetone | 1.14** | -0.32 | 0.14 | -1.58** | -0.74* | -1.32** |
| Xylitol | 0.75 | -1.13* | -0.38* | -2.03** | -0.43 | -2.19** |
| Phytosphingosine | 0.24 | -1.00* | -0.42 | -1.00** | -10.03 | -0.14 |
| Maltotriose | 0.97 | -0.09 | 0.55* | -2.00** | -11.4**8 | -1.58** |
| Tagatose | 0.46* | -2.24** | -0.38* | -1.92** | -0.01 | -4.05** |
| Galactose | 0.96* | -0.15 | -0.23 | -1.15** | -0.48 | -2.12** |
| Glucose | 0.51 | -1.81** | -0.58 | -1.58** | -0.58 | -1.58** |
| Fucose | 0.51 | -1.26** | -0.39 | -1.74** | -0.12 | -2.70** |
| Ribose | 0.40 | -1.25** | -0.68** | -0.78** | -0.57 | -2.28** |
| Mannose | 0.33 | -5.82** | -0.24 | -3.05** | 0.15 | -4.71** |
| Fructose | 0.40 | -2.20** | -0.45** | -2.00** | -0.46 | -4.05** |
| Sucrose | 0.84 | 0.24 | -1.49 | -1.07 | -1.63** | -7.17** |
| Myo-inositol | 0.91** | 1.06** | -0.67 ** | -0.18 | 0.29 | -0.42 |
| 4-aminobutyric acid | 0.21 | -1.77** | 0.05 | -1.76** | 0.02 | -1.90** |
| 5-aminovaleric acid | 0.08 | -0.56 | -1.18* | -2.70** | -0.83 | -2.70* |
| Galactonic acid | 1.08 | -0.48 | -0.67 | -1.32 | -0.81 | -3.45** |
| Saccharic acid | 0.58 | -0.39 | -0.73** | -1.42** | -0.40 | -2.00** |
| Threonic acid | 0.20 | -0.77* | -1.10 | -1.91* | -1.20** | -2.88** |
| 4-hydroxy-3-methoxybenzoic acid | 1.10 | -1.58** | -0.68 | -1.32* | -0.04 | -2.58** |
| Digalacturonic acid | 0.50 | -0.12 | -0.62** | -1.00* | -9.89 | 0.00 |
| Glycolic acid | 0.53 | 0.64 | -1.03** | -0.58** | -0.91** | -0.58* |
| Gluconic acid | 0.64 | 1.85** | -1.23* | 0.79 | -1.18** | -1.73** |
| Myristic acid | 0.29 | 0.26 | -0.77 | 0.32 | -0.24 | 0.74* |
| 3-hydroxybutyric acid | 1.22** | 0.66** | -0.08** | 1.26** | -3.81 | 1.05 |
| Glucuronic acid | 0.68** | -0.72 | 0.49 | -9.50 | 0.26 | -9.76 |
| Thymidine | 0.63** | 0.00 | -9.53** | 1.00** | 0.19 | 1.14** |
| Thymine | 4.09 | -4.08 | 5.44** | 7.02** | -0.42 | 1.15** |
| Uracil | 0.28 | 0.49 | 0.01** | 0.64** | -0.01** | 0.20 |
| Tricetin | 0.53 | 0.54 | -0.62* | -0.08 | -0.20** | 0.00** |
| 5-methoxytryptamine | 5.53** | 5.89** | -0.48** | -2.58** | -0.56* | -1.74 ** |
| Sitosterol | 0.53 | -0.15 | -9.53 | 0.37** | -1.22 | -1.00 |
| Salicylic acid | 0.63 | 0.06* | 0.23 | 0.64* | -0.09 | -8.68 ** |
| Naringin | 0.14 | 0.32 | 7.66** | 9.42** | 0.26** | -1.46 ** |
| Hydroxylamine | 0.46* | -0.68** | -1.00 | 0.25 | -0.09 | -1.09 |
| Gallic acid | 1.01* | 0.24 | 0.18 | -0.21 | 2.12** | 1.24 ** |
| Fluorene | 3.78** | 6.36** | -6.78** | 1.57* | -0.75** | -1.22 |
| Putrescine | 0.77 | -1.95 | -1.42** | -2.74 | -1.21 | -1.80 |

The fold changes were calculated using the formula log_2_^(treatment/control)^. “**” and “*” mean significant and highly significant difference, respectively; NS means non-significant difference.
