# Supplementary figures and images for: Comparison of Salt Tolerance in Soja Based on Metabolomics of Seedling Roots
Source: Front Plant Sci. 2017 Jun 23;8:1101. doi: 10.3389/fpls.2017.01101 (PMC5481370; doi:10.3389/fpls.2017.01101)

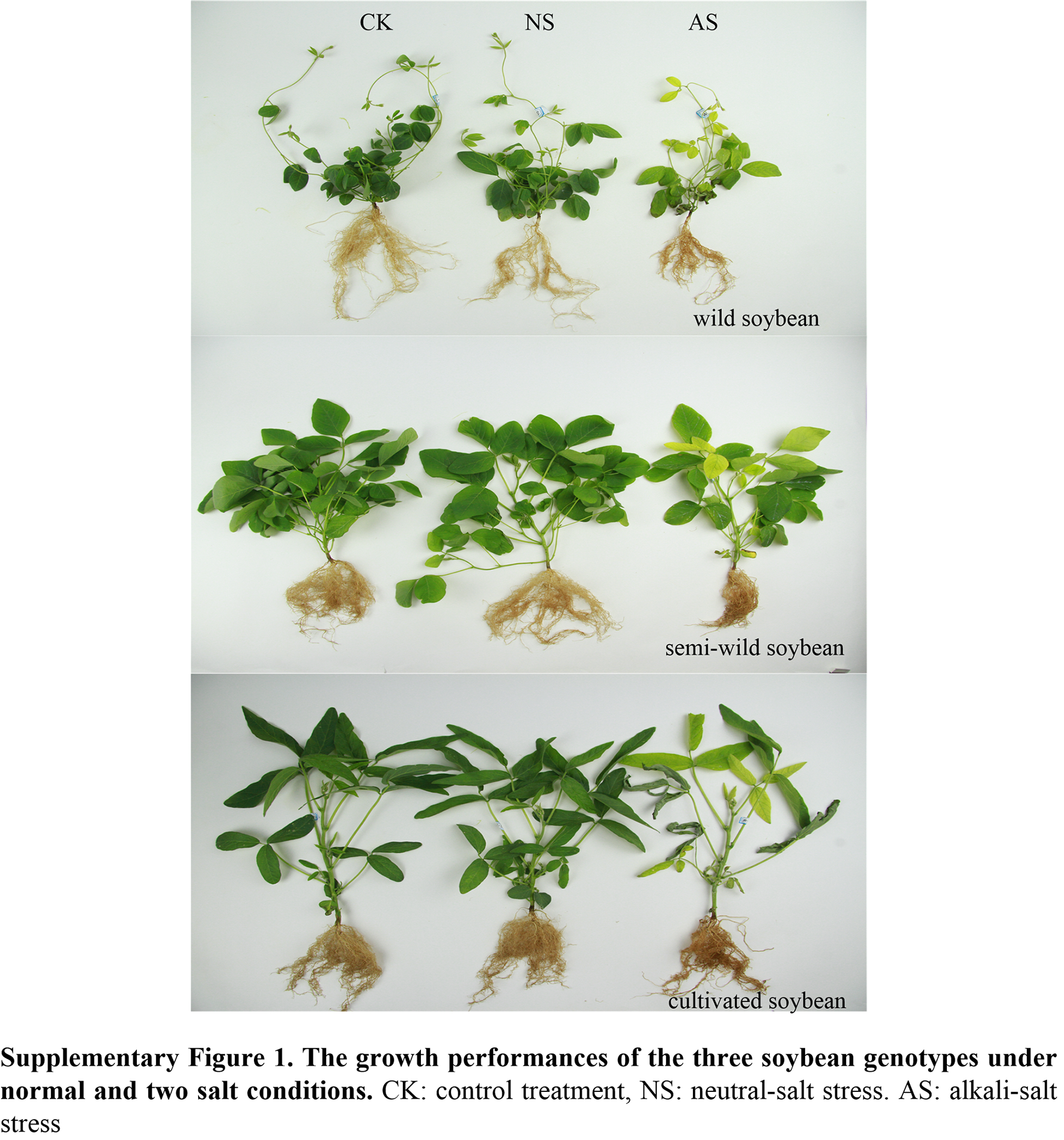

Supplement: Supplementary file 4 [file Image1.TIF]
